# Disruption of Traditional Grazing and Fire Regimes Shape the Fungal Endophyte Assemblages of the Tall-Grass *Brachypodium rupestre*

**DOI:** 10.3389/fmicb.2021.679729

**Published:** 2021-06-11

**Authors:** María Durán, Leticia San Emeterio, Leire Múgica, Iñigo Zabalgogeazcoa, Beatriz R. Vázquez de Aldana, Rosa María Canals

**Affiliations:** ^1^Grupo de Ecología y Medio Ambiente, Departamento de Agronomía, Biotecnología y Alimentación, Universidad Pública de Navarra, Pamplona, Spain; ^2^Centro Jerónimo de Ayanz, Institute on Innovation & Sustainable Development in Food Chain, Pamplona, Spain; ^3^Instituto de Recursos Naturales y Agrobiología de Salamanca (CSIC), Salamanca, Spain

**Keywords:** *Brachypodium rupestre*, disturbances, fire recurrence, culturable endophytes, *Omnidemptus graminis*, *Lachnum* sp., *Epichloë typhina*

## Abstract

The plant microbiome is likely to play a key role in the resilience of communities to the global climate change. This research analyses the culturable fungal mycobiota of *Brachypodium rupestre* across a sharp gradient of disturbance caused by an intense, anthropogenic fire regime. This factor has dramatic consequences for the community composition and diversity of high-altitude grasslands in the Pyrenees. Plants were sampled at six sites, and the fungal assemblages of shoots, rhizomes, and roots were characterized by culture-dependent techniques. Compared to other co-occurring grasses, *B. rupestre* hosted a poorer mycobiome which consisted of many rare species and a few core species that differed between aerial and belowground tissues. Recurrent burnings did not affect the diversity of the endophyte assemblages, but the percentages of infection of two core species -*Omnidemptus graminis* and *Lachnum* sp. -increased significantly. The patterns observed might be explained by (1) the capacity to survive in belowground tissues during winter and rapidly spread to the shoots when the grass starts its spring growth (*O. graminis*), and (2) the location in belowground tissues and its resistance to stress (*Lachnum* sp.). Future work should address whether the enhanced taxa have a role in the expansive success of *B. rupestre* in these anthropized environments.

## Introduction

The study of the plant microbiome is a powerful tool that contributes to the understanding and prediction of the resilience of plant communities to global change (Vandenkoornhuyse et al., 2015). In extreme environments, where ecosystems are fragile and very sensitive to changes, the plant microbiota is crucial to understand the adaptive capacity of plant communities. Low temperatures, soil acidity, low rates of mineralization, and low nutrient availability are some of the common traits of artic and alpine ecosystems, where the study of the composition and function of the plant–microbial consortium has been addressed in the last decades (Li et al., 2012; Poosakkannu et al., 2014; Rudgers et al., 2014; Bråthen et al., 2015; Kotilínek et al., 2017).

Current research reports that for a particular ecosystem, the composition and diversity of the fungal microbiome of a plant host depends on its potential for carbon provision (plant abundance and biomass), on the fungal propagule availability, and on environmental factors, mainly climate, rather than on the taxonomy of the plant host (Higgins et al., 2014; Ranelli et al., 2015; Glynou et al., 2016; Kivlin et al., 2019). The first factor, potential for carbon provision, gives support to the resource-diversity hypothesis, the highest plant carbon determines the highest guest diversity (Wang et al., 2020), and this effect is modulated by the richness and diversity of the neighboring host plants and their own fungal populations (Hiiesalu et al., 2017; Vannier et al., 2020). Regarding the climate, the historical and current precipitation regime is known to play a key role in fungal endophyte composition (Lau et al., 2013; Liu et al., 2017), and its consequences in a scenario of climate change are a growing subject of study in recent years (Giauque and Hawkes, 2016; Koide et al., 2017; Slaughter et al., 2018).

In addition to the climate change, plant communities in some cold environments are being affected by other significant mechanisms of change (Pauchard et al., 2009). Many high altitude grasslands undergo profound changes due to the disruption of the historical regime of disturbances that created and preserved them (Lasanta-Martínez et al., 2005; Komac et al., 2013). Fire and herbivory are two crucial disturbances shaping the landscape of natural grasslands worldwide (Archibald et al., 2005; Anderson and Hoffman, 2007). Extensive grazing by livestock in highlands has sharply declined in Europe in the last decades. Grazing is considered the major driver of plant guild composition and diversity in grassland communities (Milchunas et al., 1988; Canals and Sebastia, 2000; Frank, 2005; Eldridge and Delgado-Baquerizo, 2018). Grazing has profound effects on soils, promoting root exudation and carbon flow exchange to the rhizosphere (Dawson et al., 2000; Hamilton and Frank, 2001), enhancing soil microbial biomass and activating the soil biogeocycles (Bardgett and Wardle, 2003; Shaw et al., 2016), increasing and redistributing nutrients availability (Augustine et al., 2003; Liu et al., 2017) and affecting soil aeration by compaction (Jing et al., 2014). Through these effects on plants and soils, grazing may affect the structure of the fungal microbiome of plants (Schulz and Boyle, 2005; Wang et al., 2020).

Regarding fire, some high-altitude areas are currently experiencing an intensified regime of anthropic fires to reduce shrub encroachment and biomass build-ups caused by the relaxation of grazing activities (Köhler et al., 2005; Múgica et al., 2018). The effects of prescribed fires on soil properties have been documented in a range of contrasted habitats (Alcañiz et al., 2018), including high-altitude grasslands (in the Pyrenees, Armas-Herrera et al., 2016; San Emeterio et al., 2016). Although prescribed burnings do not reach the high temperatures of wildfires, their short and mid-term effects (thermal shocking, aboveground plant combustion, ash deposition, nutrient mineralization…) may drive profound changes in the plant community composition if the fire regime increases in recurrence (Uys et al., 2004). Regarding microbial soil communities, even low-intensity fires may depress soil microbial biomass (Múgica et al., 2018), with complex consequences on the C and N cycles (Soong and Cotrufo, 2015; Shaw et al., 2016; Pellegrini et al., 2020). The composition of soil fungal communities has been documented to be disrupted by fires as well (Artz et al., 2009; Egidi et al., 2016; Semenova-Nelsen et al., 2019). Few studies have addressed the effects of fire on plant fungal assemblages (Bellgard et al., 1994; Eom et al., 1999; Mataix-Solera et al., 2002), although changes in species richness have been detected in foliar endophytes of burned trees (Huang et al., 2016), and specific plant-fungal mutualisms and pyrophilous fungal species have been described (Baynes et al., 2012; Raudabaugh et al., 2020).

The western Pyrenees encompass large areas affected by the decoupling of traditional fire and grazing practices. As a result of decreasing herbivore pressure and increasing burnings, a native perennial grass is expanding. *Brachypodium rupestre* (Host) Roem. & Schult (Schippmann, 1991; Schippmann and Jarvis, 1988) (=*B. pinnatum* subsp. *rupestre* (Host) Schübl. & Martens according to some authors), (Aizpuru et al., 1999), dominates grasslands and causes a severe loss of sympatric species (Canals et al., 2014; Canals, 2019), a phenomenon also observed in other European mountain ranges (Buckland et al., 2001; Holub et al., 2012; Tardella et al., 2018). In this natural setting, many questions arise concerning the response of the plant holobiont to the changed disturbance regime. To what extent the composition of the *B. rupestre* mycobiome responds to the altered disturbance regime? Does the species harbor a specific fire-adapted mycobiome in frequently burnt areas? Or on the contrary, is there sufficient inertia to maintain a similar mycobiome in a common climatic environment (cold temperatures, high rainfall and humidity), independently of the current disturbance regime? Since taxon-specific endophytes (such as *Epichloë* sp.) may confer a higher adaptive advantage in stressful situations than broadly distributed non-systemic endophytes (Hill et al., 1989; Malinowski and Belesky, 2000; Pereira et al., 2019; Harrison and Griffin, 2020), is the mycobiome of the plant responding to these expectations?

The main objective of this research was to characterize the culturable fungal endophyte community of *B. rupestre* across a well-defined gradient of disturbance. The gradient encompassed sites with different burning recurrences and levels of herbivory, which have led to different patterns of grassland structure and diversity. We also studied the fungal endophyte community of the aerial tissues of the most frequent companion species of *B. rupestre* in grasslands, *Festuca rubra* and *Agrostis capillaris*. The purpose was to estimate whether the major diversity of coexisting plant species (and potential fungal propagules) in diverse grasslands had a positive influence in the fungal assemblage of *B. rupestre*.

## Materials and Methods

### Study Site and Sampling Design

**FIGURE 1:**
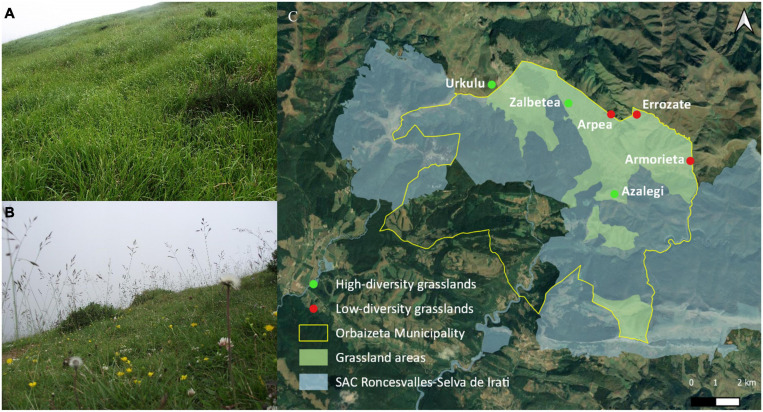
Locations selected for collecting plants. Red points represent low-diversity grasslands and green points high-diversity grasslands **(C)**. Pictures of the *Brachypodium rupestre* invaded **(A)** and non-invaded **(B)** areas.

The study area was located in the Aezkoa Valley in the western Pyrenees (43°3′ N1°13′ W; 800–1900 m.a.s.l.), and occupies an area of 198 Km^2^. Because of the influence of the Atlantic ocean (55 km away in a straight line) the weather is cold and snowy during winter, and mild and foggy during summer. The mean annual temperature is 9.3°C and mean annual precipitation is 1856 mm, according to data collected during 1989–2019 at the nearest climatic station, Irabia at 822 m.a.s.l.^1^. Soils are mainly derived from sandstones and calcareous clays, acidic with high organic matter and loamy or clay-loamy textures. The landscape is a mosaic of beech forests, gorse shrublands dominated by *Ulex gallii* Planch and *Erica vagans* L., and grasslands dominated by perennial grasses such as *Festuca rubra gr.*, *Agrostis capillaris* L., *Brachypodium rupestre* (Host) Roem. & Schult, *Danthonia decumbens* (L.) and *Avenula sulcata* (J. Gay ex Boiss) Dumort. Other species are *Galium saxatile* L., *Potentilla erecta* (L.) Räeusch, *Potentilla montana* Brot., *Hypochaeris radicata* L., and the legume *Trifolium repens L.* The area is part of the European Protected Areas Network (Natura 2000) and was declared Special Area of Conservation (Roncesvalles-Selva de Irati, code ES0000126) in 2011 (Figure 1).

High-altitude grasslands are extensively grazed from May to October by sheep, cows and horses. Domestic herbivores have been present in the area since the Neolithic, as numerous prehistoric pastoral remains indicate. Where an appropriate extensive summer grazing exists, grasslands constitute high-diversity communities that are burned every 6–7 years by the bush-to-bush traditional practice to control localized shrub resprouting (San Emeterio et al., 2016). However, in the last decades, the number of grazers has sharply declined due to socio-economical changes experienced in the valleys, and the use of repeated winter burnings to control the build-up of ungrazed biomass has increased (Durán et al., 2020). Nowadays, many areas are burned recurrently, every 1–2 years (Canals et al., 2017). Due to the frequent burnings and the lack of grazing, the grasslands are far less diverse, favored by the expansion and dominance of *B. rupestre*, which grows in dense and tall clumps. Consequently, the current open landscape is a combination of grassland communities with different degrees of cover by *B. rupestre*, which accurately reflects the level of herbivory and the burning recurrence.

Based on the cartographic information provided by the Management Plan of the area (Ferrer and Canals, 2008), we selected six grassland communities where *B. rupestre* was present at different coverages: three areas had above 75% cover and three areas under 25% cover (Table 1 and Figure 1). The detailed floristic communities of the area are available in Durán et al., 2020. At each location, 40 turfs (400 cm^3^) of *B. rupestre* (including shoots, rhizomes, roots and rhizosphere) were collected in summer 2018. We established a distance of ca 30 m among collected plants to avoid clonal individuals (Baba et al., 2012). In the three areas of diverse grasslands, 40 turfs of *Festuca rubra* and 40 turfs of *Agrostis capillaris* were also collected. Sampling points were georeferenced and the sampling grid covered ca 2.5 ha per location (Supplementary Figure 1). In total, 480 turfs were collected, placed in seedbed trays and transported to the UPNA laboratory. The plants in the turfs were processed for the isolation of fungi from shoots, rhizomes and roots in the same week.

**TABLE 1:** Characteristics of the six locations selected.

**Location**		**Fire recurrence**	**Grazing level**	***B. rupestre* cover (%)**	**Plant diversity**
1	Arpea	High 1–2 years	Low to non-existent	>75%	Very low
2	Errozate				
3	Armorieta				
4	Urkulu	Low 6–7 years	Moderate	<25%	High
5	Zalbetea				
6	Azalegi				

### Isolation and Identification of Fungi

We isolated fungi from shoots, rhizomes and roots of *B. rupestre* and from shoots of *F. rubra* and *A. capillaris*. Leaf sheaths and stems were cut into fragments of ca 5 mm, and surface-disinfected by immersion in a solution of 20% commercial bleach (1% active chlorine) containing 0.02% Tween 80 (v:v) for 10 min, and finally rinsed with sterile water. The fragments of rhizomes and roots were surface-sterilized with the same bleach-Tween 80 solution, but then treated with an aqueous solution of 70% ethanol for 30 s, and a final rinse with sterile water. About 10–12 tissue fragments of the same individual were plated in a Petri dish with potato dextrose agar (PDA) containing 200 mg/L of chloramphenicol to avoid the growth of endophytic bacteria. Petri dishes, kept at room temperature and ambient light, were checked daily for mycelium growth during 5 weeks. When mycelium emerged from a tissue fragment, a small amount was transferred to a new Petri dish to obtain a culture.

Isolated fungi were grouped into morphotypes according to their morphological characteristics (colony color, exudates, growth type, and mycelium appearance). One or more isolates of each morphotype were genotyped for taxonomic purposes. To do this, a small amount of mycelium was scratched from the isolate culture and its DNA extracted using the Phire Plant Direct PCR Kit (Thermo Fisher Scientific). The ITS1, 5.8s and ITS2 regions were amplified using ITS4 and ITS5 primers (White et al., 1990). PCR amplification was done at 98°C for 5 min, followed by 35 cycles of 95°C for 5 s, 54°C for 5 s, 72°C for 20 s, and a final phase of 72°C for 1 min. Amplicons were purified (Favor Prep^TM^ Plant Genomic DNA Extraction Mini Kit, Favorgen) and sequenced by the Sanger method at an external sequencing service (STABvida).

Because the range of intraspecific variation in ITS sequences is unknown for most fungal species (Taylor et al., 2000), DNA sequences were clustered using the CD_HIT program (Li and Godzik, 2006; Huang et al., 2010) and those with 97% or more similarity were considered to belong to the same taxon. A representative sequence of each cluster was selected and used to search the database of the ITS region from fungi type and reference material (Schoch et al., 2014) at the National Center for Biotechnology Information (NCBI) using the BLAST algorithm. In addition, the UNITE database of fungal nucleotide sequences was used as a complement for sequences without type specimens in NCBI. We also used the database FUNGuild (Nguyen et al., 2016) to get information on the ecological guild of each taxon, and to estimate for their possible functional roles.

### Data Analyses

To evaluate the efficiency of our sampling effort for measuring species richness, species accumulation curves were estimated with all the species, and also excluding singletons, species that appeared only once (Vegan package, Oksanen et al., 2017). We calculated the incidence of all endophyte species within each type of tissue (shoots, rhizomes and roots), grassland type (low and high-diversity) and host plant species, and determined richness and Shannon and Simpson diversity indexes (Vegan package, Oksanen et al., 2017). Differences in species richness and diversity indexes of *B. rupestre* endophytes were analyzed using two-way ANOVA with tissue and grassland type as fixed factors, and permutation tests was used to compare diversity indexes of *B. rupestre*, *F. rubra*, and *A. capillaris* shoots (Coin package, Hothorn et al., 2008).

Venn diagrams were used to represent the taxa shared among grassland types, plant tissues and grass species (Euler package, Chen and Boutros, 2011). Frequencies were calculated from the matrix of presence/absence of isolates and permutational analyses of variance (PERMANOVA) were used to evaluate the variability of the fungal endophyte assemblages of *B. rupestre* between grassland types and among plant tissues. For that purpose, Adonis function was used (Vegan package, Oksanen et al., 2017). Distances were calculated using Bray–Curtis dissimilarities, set the number of permutations to 9999 and constrained the permutations within each location. Since PERMANOVA analyses are very sensitive to heterogeneity of multivariate dispersions, homogeneous dispersion between treatment groups was tested using the betadisper function.

To identify which species were characteristic of a particular tissue and grassland type, the indicator species tests in the Labsdv package was used (Roberts, 2019). The indicator value of a species (indval) measures the fidelity and relative abundance of the species in a particular situation (Dufrêne and Legendre, 1997). General Linear Mixed Models (GLMM) was used to determine whether the indicator species for a particular grassland type presented differences in the probability of incidence among grassland types. The grassland type was included as the fixed factor, the location as the random factor, using a binomial distribution. In addition, GLMM was used to analyze whether the probability of incidence of some mycobiome core species from the aboveground tissues presented differences among host plant species. Host plant species was included as the fixed factor, location as the random factor, using a binomial distribution. GLMM’s were done using the lme4 package (Bates et al., 2015).

## Results

### Isolation and Identification of Fungal Endophytes

We plated ca 10000 tissue fragments of *B. rupestre* (shoots, rhizomes and roots), *F. rubra* (shoots) and *A. capillaris* (shoots) in 960 culture media plates (10–12 fragments per plate). We obtained 1151 isolates (ca 190 isolates per location) which were classified into a total of 95 morphotypes. One or more isolates of each morphotype were sequenced, obtaining 116 sequences. After the sequence clustering process, 61 different sequence types remained, which were identified into 53 different taxa using the NCBI and UNITE databases. Twenty-two of them were classified to species rank, 20 to genus, 6 to family, 3 to order, and the remaining two to class rank (Supplementary Table 1 contains the complete list of taxa identified and their accession number).

### Diversity of the *B. rupestre* Mycobiome

**FIGURE 2:**
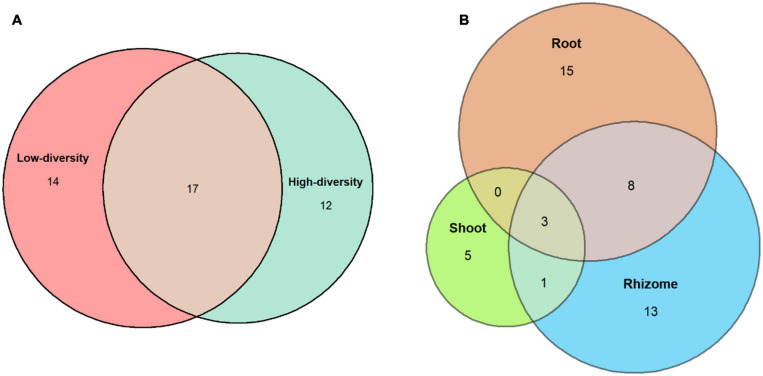
Venn’s diagrams indicating the number of shared fungal species across grassland types **(A)** and tissues **(B)**.

Fungi were isolated from 58% of the *B. rupestre* plants in low-diversity grasslands and from 43% in high-diversity grasslands (Table 2). Endophyte incidence varied among tissues: 37% of shoots, 52% of rhizomes and 64% of roots harbored fungi. Forty-five different taxa were identified in *B. rupestre*. Endophyte species richness in *B. rupestre* shoots, rhizomes and roots were 9, 25, and 26 taxa, respectively. Endophyte species richness in *B. rupestre* growing in low-diversity grasslands ranged from 9 to 22, and from 12 to 18 taxa in high-diversity grasslands (Table 2). Roots and rhizomes shared 23.9% of the species, whereas shoots with rhizomes and with roots shared 8.7 and 6.5%, respectively, all tissues had only 6.5% of all species in common (Figure 2). *B. rupestre* plants in low and high-diversity grasslands shared 39.1% of the species.

**TABLE 2:** Number of plants collected, final plates (taking into account the contaminations), fungal endophyte incidence (% plants), and number of fungal species in *Brachypodium rupestre*, all of that according to tissue and location.

	**Tissue**	**Number of plants**	**Number of plates**	**Endophyte incidence (% plants)**	**Number of fungal species**
	Shoot	240	231	37	9
	Rhizome	240	213	52	25
	Root	240	228	64	26
	Total	240	672		45
***Cover***	**Location**				
Low-diversity grasslands	*Arpea*	40	107	59	21
	*Errozate*	40	112	60	9
	*Armorieta*	40	110	56	22
	Total	120	329		32
High-diversity grasslands	*Urkulu*	40	114	42	12
	*Zalbetea*	40	114	49	18
	*Azalegi*	40	115	39	16
	Total	120	343		30

The most frequent species were *Albotricha* sp., *Lachnum* sp. B, *Omnidemptus graminis*, and Mollisiaceae sp. (Table 3). *Omnidemptus graminis* was more frequent in shoots than in rhizomes and absent in roots, whereas *Albotricha* sp., *Lachnum* sp. B, and Mollisiaceae sp. were more frequent in belowground tissues than in shoots. In addition to the Mollisiaceae sp. taxon, other dark septate endophytes (DSE) such as *Cadophora* sp., *Microdochium bolleyi*, *Microdochium neoqueenslandicum*, and *Periconia* sp. were found, although with a low incidence. Members of the *Clavicipitaceae* family, such as *Metapochonia bulbillosa*, *Metarhizium carneum*, and *Epichlöe typhina* in shoots and rhizomes also occurred. The frequencies of the remaining species were under 2% (Supplementary Table 2). Three trophic types (pathotroph, saprotroph, and symbiotroph) were found in the mycobiome of *B. rupestre*. The most common guilds were plant pathogens, endophytes and undefined saprotrophs. However, a remarkable number of taxa were animal pathogens (*Clonostachys rosea*, *Tolypocladium album*, and *Trichoderma koningii* are described as entomopathogens, *Metapochonia bulbillosa* and *Sarocladium strictum* as nematophagous). Fungi with antimicrobial activity (*Fusarium circinatum*, *Glarea* sp., *Nemania* sp., and *Penicillium ortum*) and species with a high source of bioactive compounds (*Acremonium* sp., *Gaeumannomycella* sp., and *Lachnum* sp.) also occurred (Supplementary Table 1).

**TABLE 3:** Incidence in *Brachypodium rupestre* plants of the most abundant fungal endophyte taxa, differentiated by tissues and number of locations where they were present.

		**Low-diversity grasslands**		**High-diversity grasslands**	
**Tissue**	**Fungal endophyte**	**Incidence (%)**	**Number of locations**	**Incidence (%)**	**Number of locations**
Shoots	*Omnidemptus graminis*	46.1	3	23.3	3
	*Epichloë typhina*	2.6	1	3.4	2
	*Sarocladium strictum*	3.5	2	0	0
Rhizomes	*Lachnum* sp. B	13.9	3	8.9	2
	Mollisiaceae sp.	14.9	2	7.1	2
	*Albotricha* sp.	2.0	1	16.1	2
	*Omnidemptus graminis*	5.9	1	5.4	2
	*Metapochonia bulbillosa*	3.0	1	2.7	3
	*Fusarium circinatum*	3.0	3	1.8	1
	*Penicillium ortum*	2.0	2	2.7	1
	*Tolypocladium album*	3.0	1	0.9	1
	*Clonostachys rosea*	2.0	1	1.8	2
	*Ilyonectria robusta*	2.0	2	0.9	1
	*Microdochium bolleyi*	0	0	2.7	2
Roots	*Lachnum* sp. B	35.4	3	16.5	3
	Mollisiaceae sp.	14.2	3	14.8	3
	*Albotricha* sp.	9.7	3	19.1	2
	*Lachnum* sp. A	3.5	3	2.6	2
	*Ilyonectria robusta*	0.9	1	5.2	2
	*Glarea* sp.	1.8	2	3.5	2
	*Dictyochaeta* sp.	3.5	2	0	0
	*Acremonium* sp.	1.8	2	1.7	1
	*Fusarium circinatum*	0	0	3.5	2
	*Mollisia* sp.	2.7	1	0	0

**FIGURE 3:**
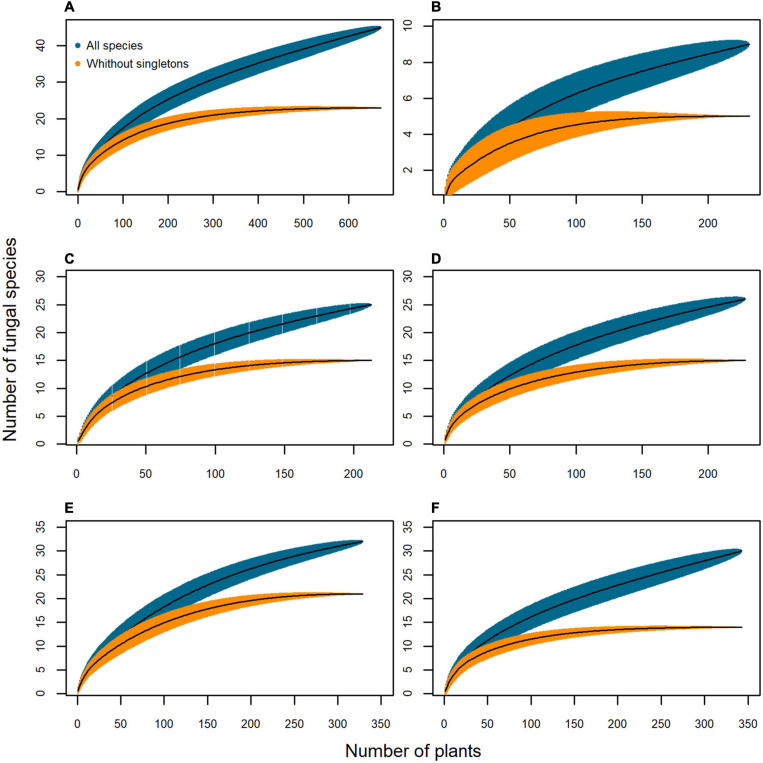
Species-accumulation curves of the mycobiota isolated from *Brachypodium rupestre* according to plant tissue and grassland type. Black line, based estimator of the total number of species; shaded zone, standard deviation; dark blue, all species included; orange, excluding singleton species. **(A)** All tissues, **(B)** Shoots, **(C)** Rhizomes, **(D)** Roots, **(E)** Low-diversity grasslands, **(F)** High-diversity grasslands.

The species-accumulation curves including all the endophyte taxa were non-asymptotic (Figure 3), but when singletons were excluded the curves reached a plateau, suggesting that an increase in the sampling effort would reveal few common taxa, and mostly rare species. Therefore, a sampling effort of 100 plants is adequate to flatten the curves of the different tissues when excluding the singletons (Figures 3B–D). A greater sampling effort would be needed to include all the tissues (Figure 3A), although low-diversity grasslands need more plants than high-diversity grasslands to characterize their endophyte richness (Figures 3E,F).

**FIGURE 4:**
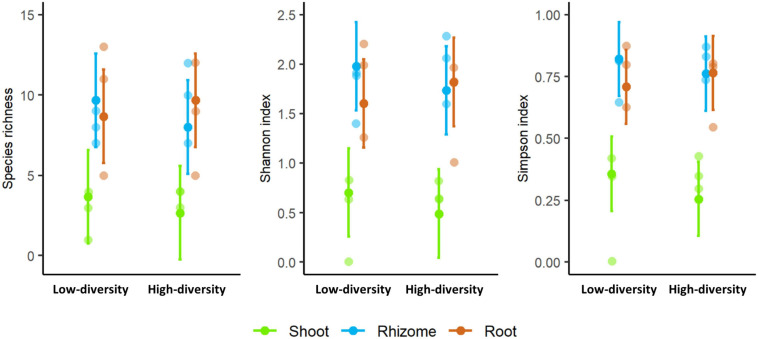
Species richness and diversity indexes (Shannon and Simpson) of the mycobiome of shoots, rhizomes, and roots of *Brachypodium rupestre* in low and high-diversity grasslands.

Species richness and diversity indexes were significantly different among tissues (*F* = 10.319, *p* < 0.001; *F* = 18.336, *p* < 0.001; *F* = 24.114, *p* < 0.001; for richness, Shannon and Simpson, respectively). Shoots showed consistently lower diversity values than rhizomes or roots in both types of grasslands (Figure 4). No significant differences in diversity were found between low and high-diversity grasslands (*F* = 0.210, *p* > 0.05; *F* = 0.180, *p* > 0.05; *F* = 0.311, *p* > 0.05; for richness, Shannon and Simpson, respectively).

### Distribution Patterns of the *B. rupestre* Mycobiome

**FIGURE 5:**
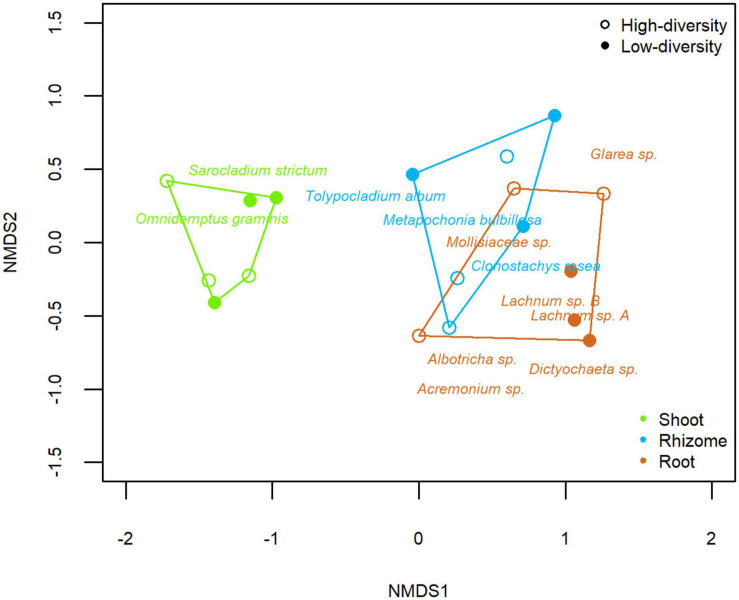
Non-metric multidimensional scaling (NMDS) of the fungal endophyte community composition of tissues of *Brachypodium rupestre* from low and high-diversity grasslands.

A homogeneous dispersion was found within different tissues (Beta-disper; *F*2,15 = 2.195, *p* = 0.146) and grassland types (Beta-disper; *F*1,16 = 0.028, p = 0.870), contrasting with the significant effects on community assemblages. PERMANOVA analysis showed that tissue and grassland type had a significant effect on the endophytic community of *B. rupestre* plants (both *p* < 0.001). Tissue explained ca 50% of the variance of the model (*r*^2^ = 0.508), whereas grassland type explained ca 5% (*r*^2^ = 0.052). The NMDS (Non-Metric Multidimensional Scaling) plot discriminated the assemblages according to the tissues and included the indicator species (Figure 5). *Omnidemptus graminis* (*p* = 0.001) and *Sarocladium strictum* (*p* = 0.032) were indicators of shoots. *Metapochonia bulbillosa* (*p* = 0.003), *Clonostachys rosea* (*p* = 0.011), and *Tolypocladium album* (*p* = 0.009) were indicators of rhizomes. And *Lachnum* sp. B (*p* = 0.001), *Albotricha* sp. (*p* = 0.001), Mollisiaceae sp. (*p* = 0.001), *Lachnum* sp. A (*p* = 0.008), *Acremonium* sp. (*p* = 0.021), and *Dictyochaeta* sp. (*p* = 0.024) were indicators of roots. Regarding the grassland type, *Albotricha* sp. (*p* = 0.001) was an indicator of high-diversity grasslands and *Lachnum* sp. B (*p* = 0.004) and *Omnidemptus graminis* (*p* = 0.003) of low-diversity grasslands.

**FIGURE 6:**
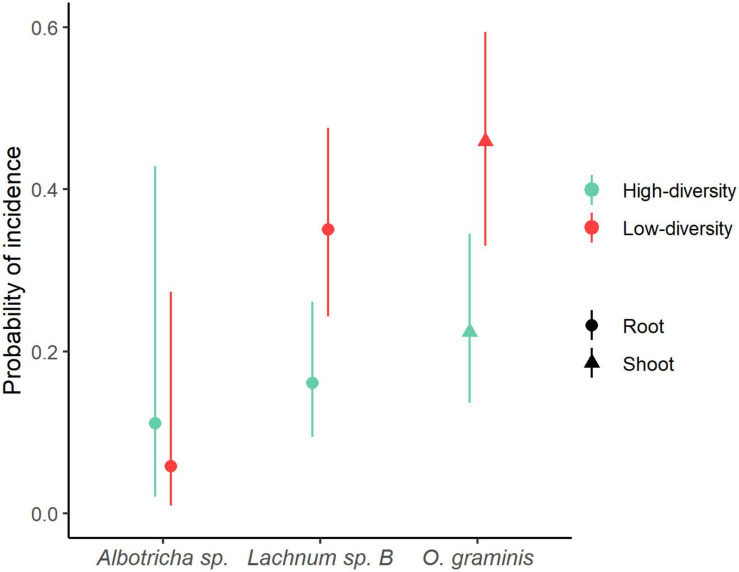
Probability of incidence of fungal indicator species of *Brachypodium rupestre* calculated with GLMM.

GLMM’s showed that *B. rupestre* plants from low-diversity grasslands had a greater probability of having their roots infected by *Lachnum* sp. B (LRT = 4.5719, *p* = 0.032) and their shoots by *Omnidemptus graminis* (LRT = 4.679, *p* = 0.030) than plants from high-diversity grasslands (Figure 6). Regarding *Albotricha* sp., the patterns were not so clear and its probability of incidence in *B. rupestre* roots was not significantly different between grassland types (LRT = 0.2849, *p* = 0.594, Figure 6).

### Aboveground Endophyte Assemblages of the Most Common Grasses in High-Diversity Grasslands

The incidence of endophytes varied among the shoots of the three grass species, ranging from 25% in *B. rupestre* and *A. capillaris* to 38% in *F. rubra*. For each plant species, a strong variability of endophyte incidence among locations was detected (Table 4). Eight taxa not observed in the *B. rupestre* survey were isolated from *F. rubra* or *A. capillaris* (Supplementary Table 1).

**TABLE 4:** Number of plants collected, number of final plates (taking into account the contaminations), fungal endophyte incidence (% plants), and number of fungal taxa per location for each grass species from high-diversity grasslands in aboveground tissues.

**Grass species**	**Location**	**Number of Plants**	**Number of plates**	**Endophyte incidence (% plants)**	**Number of fungal species**
*B. rupestre*	*Urkulu*	40	39	26	4
	*Zalbetea*	40	39	41	3
	*Azalegi*	40	38	8	1
	Total	120	116		5
*F. rubra*	*Urkulu*	40	39	51	7
	*Zalbetea*	40	38	8	1
	*Azalegi*	40	40	55	5
	Total	120	117		10
*A. capillaris*	*Urkulu*	40	40	38	7
	*Zalbetea*	40	40	20	8
	*Azalegi*	40	39	18	6
	Total	120	119		16
					

**FIGURE 7:**
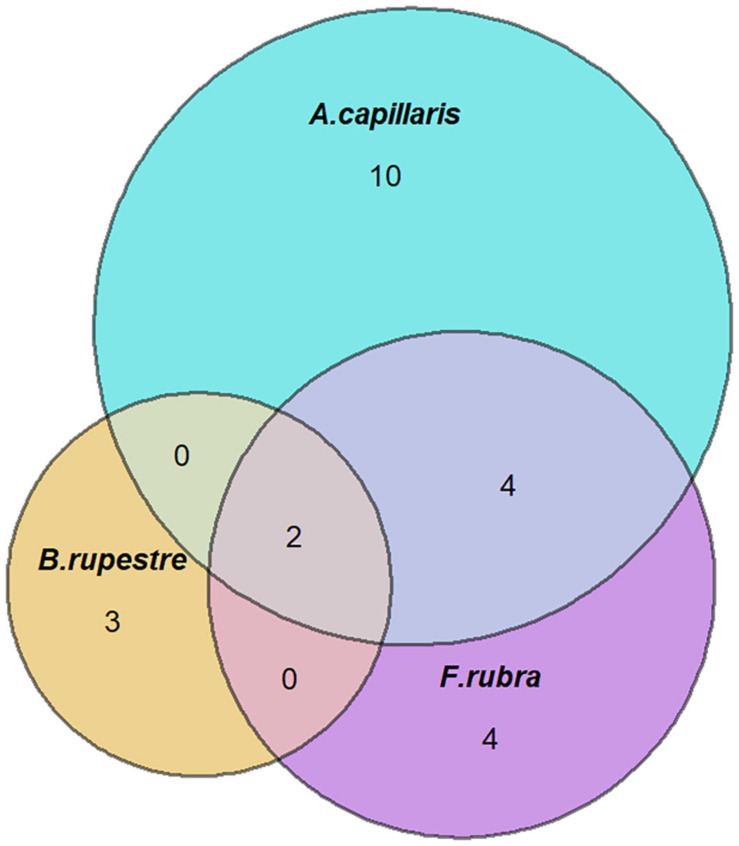
Venn’s diagrams indicating the number of shared fungal endophyte species among grass species.

A total of 23 taxa were isolated from the shoots of *B. rupestre*, *F. rubra* and *A. capillaris* (Supplementary Table 3). Endophyte species richness in *B. rupestre, F. rubra*, and *A. capillaris* shoots were 5, 10, and 16, respectively (Table 4). *F. rubra* and *A. capillaris* shared more endophyte species between them than *B. rupestre* with either (Figure 7). The three grasses had two endophyte species in common, *Lachnum* sp. B and *Omnidemptus graminis*. The most frequent endophyte species in *B. rupestre* shoots were *Omnidemptus graminis* and *Epichloë typhina*, in *F. rubra Epichlöe festucae* and Mollisiaceae sp., and in *A. capillaris Lachnum sp.* B, *Epichloë baconii* and Mollisiaceae *sp.* (Table 5).

**TABLE 5:** Percentage of incidence of dominant fungal endophyte species in shoots of three representative grasses of high-diversity grasslands.

		**Incidence (%)**		
**Grass**	**Fungal endophyte**	**Urkulu**	**Zalbetea**	**Azalegi**
*B. rupestre*	*Omnidemptus graminis*	23.1	38.5	7.9
	*Epichloë typhina*	2.6	7.7	0
*F. rubra*	*Epichloë festucae*	23.1	0	50.0
	Mollisiaceae sp.	20.5	0	2.5
	*Albotricha sp.*	0	7.9	2.5
	*Alfaria dandenongensis*	7.7	0	0
	*Omnidemptus graminis*	0	0	5
*A. capillaris*	*Lachnum* sp. B	15.0	0	0
	*Epichloë baconii*	7.5	0	5.1
	Mollisiaceae sp.	12.5	0	0
	*Alfaria dandenongensis*	5.0	2.5	0
	*Ilyonectria robusta*	2.5	0	2.6
	*Albotricha sp.*	5.0	0	0

**FIGURE 8:**
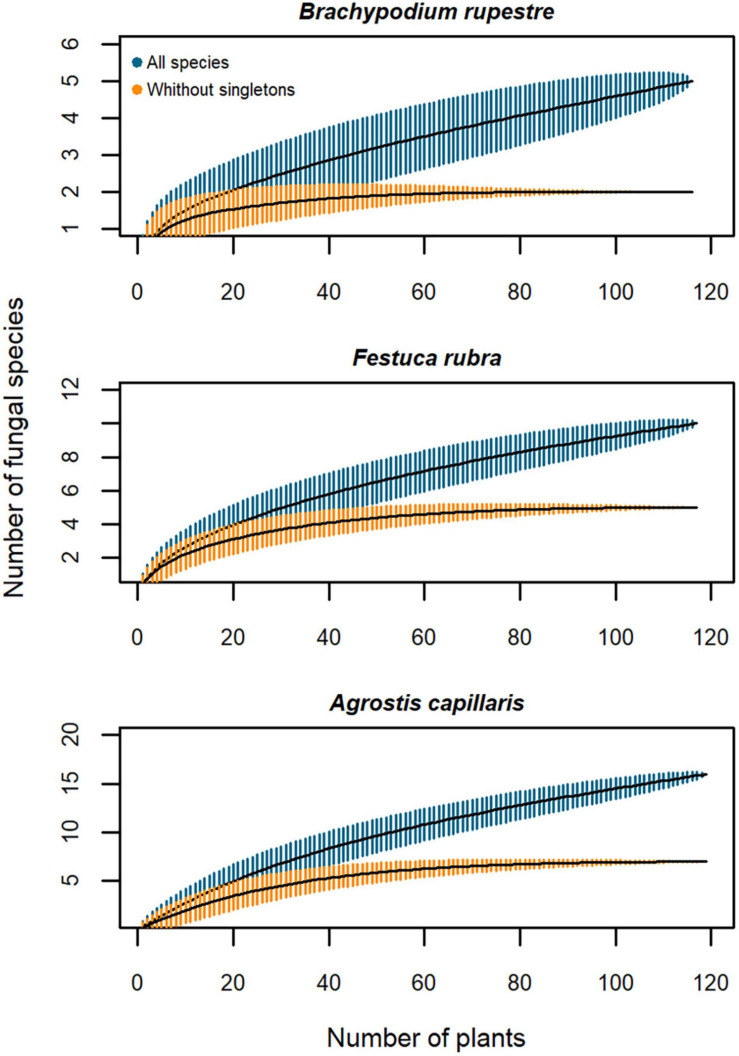
Species-accumulation curves of fungal endophytes from *Brachypodium rupestre*, *Festuca rubra* and *Agrostis capillaris* shoots from high-diversity grasslands. Black line, based estimator of the total number of species; shaded zone, standard deviation; dark blue, all species included; orange, excluding singleton species.

When including all endophyte species, the species-accumulation curves of the three host grasses were non-asymptotic (Figure 8), but when removing singletons the curves flattened with a sampling effort of 50 plants for *B. rupestre* and 60 plants for *F. rubra* and *A. capillaris* in high-diversity grasslands (Figure 8).

**FIGURE 9:**
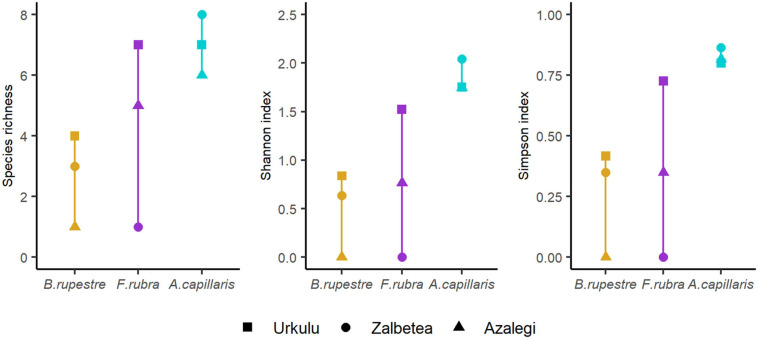
Species richness and diversity indexes (Shannon, Simpson) of the mycobiome of shoots from three representative grasses of high-diversity grasslands from three locations.

The mycobiome of *A. capillaris* appeared to be more rich and diverse (averaged per site richness_AC_ = 7.0, Shannon_AC_ = 1.85, Simpson_AC_ = 0.83) than that of *F. rubra* and *B. rupestre* (averaged richness_FR_ = 4.3, Shannon_FR_ = 0.76, Simpson_FR_ = 0.36; averaged richness_BR_ = 2.7, Shannon_BR_ = 0.49, Simpson_BR_ = 0.26). However, the high variability among locations and the size of the sample (Figure 9) did not allow to detect significant differences in richness and diversity indexes among grasses in the permutations tests (richness, *t* = 1.7876, *p* = 0.1737; Shannon, *t* = 2.0697, *p* = 0.09614; Simpson, *t* = 2.0193, *p* = 0.1076).

**FIGURE 10:**
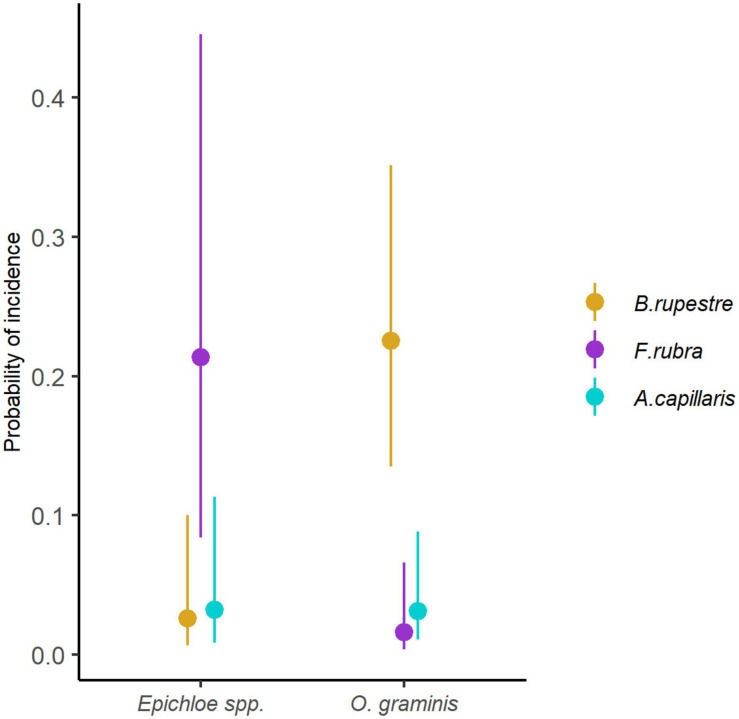
Probability of incidence of *Epichloë* spp. and *Omnidemptus graminis* in the aboveground tissues of the most common grasses of high-diversity grasslands.

*Omnidemptus graminis* and *Epichloë typhina* were species indicators of *B. rupestre* (*p* = 0.001; *p* = 0.008), whereas *Epichloë festucae* was a species indicator of *F. rubra* (*p* = 0.001) and *Epichloë baconii* of *A. capillaris* (*p* = 0.009). The GLMM showed that the probability of incidence of *Omnidemptus graminis* significantly varied among species (LRT = 38.194, *p* < 0.001), and had a higher probability of incidence in *B. rupestre*, despite its presence in the other grasses (Figure 10). On the contrary, *Epichloë* species were specific of each grass (*E. typhina* in *B. rupestre*, *E. festucae* in *F. rubra* and *E. baconii* in *A. capillaris*), but the probability of *Epichloë* infection was higher in *F. rubra* than in the rest of grasses (LRT = 34.581, *p* < 0.001; Figure 10).

## Discussion

### Shoot Fungal Endophyte Assemblages of the Most Common Grasses

The richness and diversity of the mycobiomes from this survey was relatively low compared to other studies in grasses (Sánchez-Márquez et al., 2008, 2010; Pereira et al., 2019). The study of fungal endophytes is highly method-dependent (Hyde and Soytong, 2008). In this research, we applied a conservative protocol, with along sterilization process -i.e., 10 min vs. 6 min in Pereira et al. (2019)-, and discarding the mycelia growing in the first day, which might influence the final counts of species (Burgdorf et al., 2014).

Despite the low richness reported, interesting patterns emerged from the shoot assemblages of the grasses. *B. rupestre* hosted the poorest mycobiome, half of that present in *F. rubra*, and one third of that of *A. capillaris.* Only two of 23 endophyte species identified were common to all grasses (*Omnidemptus graminis* and *Lachnum* sp. B). Recent studies indicate that plant identity influences foliar fungal assemblages more than expected from previous literature (Kivlin et al., 2019). Since the three grasses are sympatric and share an identical abiotic environment (i.e., climate, soil, location), other factors might define their mycobiomes. Plant abundance and capacity for carbon provision (*B. rupestre* develops more biomass but it is generally less abundant in diverse grasslands than the other grasses), potential interactions among fungal species (i.e., *Omnidemptus graminis*, which is abundant in *B. rupestre*, can induce host defense against other fungi (Schulz and Boyle, 2005; Constantin et al., 2020) and the different degree of herbivory (*B. rupestre* is less palatable to livestock than *A. capillaris* and *F. rubra*), may play a definite role in the structure of fungal assemblages.

Regarding systemic endophytes, the three grasses were infected by species of the genus *Epichloë*. *E. typhina, E. baconii*, and *E. festucae* are highly specific for *B. rupestre*, *A. capillaris*, and *F. rubra*, respectively (Leuchtmann et al., 2014; Saikkonen et al., 2016). *Epichloë* species are known to produce long-term, systemic infections and have a narrow range of hosts, limited to a genus or to related genera (Schardl et al., 2009; Schirrmann et al., 2015). In the area of study, the incidence of *Epichloë* was variable depending on species and locations, and symbioses with *E. festucae* were by far the most abundant (up to 50% incidence in one location) compared to *E. typhina* and *E. baconii* (less than 8% incidence in all locations). This is in accordance with previous research showing that the incidence of *Epichloë*-grass infections change depending on the associations (Leuchtmann and Schardl, 1998). *E. festucae* mainly reproduces asexually by vertical transmission to seeds, while in *E. typhina* and *E. baconii* seed transmission is absent (Leuchtmann et al., 2014). *B. sylvaticum*, a species close to *B. rupestre*, is very common in forest undergrowth, and displays high levels of infection by *E. sylvatica*, a seed-transmitted species (Meijer and Leuchtmann, 1999; Brem and Leuchtmann, 2001). The results suggest that the capacity of systemic endophytes to infect host seeds and transmit vertically, which is the most common reproductive mechanism in *Epichloë* species, leads to higher levels of incidence than in sexual, horizontally transmitted *Epichloë* species. This makes sense because in vertically transmitted endophytes reproductive fitness is intimately tied to that of their hosts (Saikkonen et al., 2002). As a consequence, mutualisms are expected to be more successful and intense in these situations. Of the three *Epichloë* species identified, *E. festucae* has been extensively studied for its capacity to establish successful symbioses in the most extreme environments (Vázquez-de-Aldana et al., 2013b; Zabalgogeazcoa et al., 2013; Leinonen et al., 2019; Pereira et al., 2019), and for the variety of advantages that confers to the host plant (i.e., tolerance to stress, resistance to herbivores, plant fitness) (Malinowski and Belesky, 2000; Zaurov et al., 2001).

### Fungal Tissue Assemblages and Core Mycobiome of *B. rupestre*

Roots and rhizomes of *B. rupestre* displayed different endophyte assemblages than aboveground tissues, had the greatest percentages of fungal infection (Figure 5), and a significantly high richness and diversity of taxa. Previous studies have shown that belowground tissues have greater endophyte diversity than aerial tissues, and this could be related to the level of tissue exposition to fungal inoculum (Sánchez-Márquez et al., 2010; Harrison and Griffin, 2020). In *B. rupestre*, as in many perennial grasses, most aboveground tissues die and renew annually, while belowground tissues such as rhizomes, survive and consequently have a larger time frame for fungal reinfection.

As in other grass species, the mycobiome of *B. rupestre* was constituted by a few core species and many rare species (Comby et al., 2016; Ofek-Lalzar et al., 2016; Pereira et al., 2019; Sun et al., 2020). Only two species were found in more than 20% of the *B. rupestre* plants and in most locations, *Omnidemptus graminis* in shoots and rhizomes and *Lachnum* sp. B in roots and rhizomes. *O. graminis* is a member of the Magnaporthaceae that has been described recently (Hernández-Restrepo et al., 2019). Although some Magnaporthaceae strains have shown plant-growth promoting activity (Yuan et al., 2010; Changyeol et al., 2017), the best known members of this family are pathogens of grasses, associated to roots (*Gaeumannomyces graminis* and *Magnaporthe poae)* and shoots (*Magnaporthe oryzae*) (Illana et al., 2013). *M. oryzae* is a hemibiotrophic fungus that causes the rice blast disease. This species switches from a biotrophic growth phase -it feeds from the host plant without killing its cells- in early infection to a necrotrophic stage (Kankanala et al., 2007). According to Talbot et al. (1997), the switch between phases may be triggered by the lack of nutrients within the host cell. In this survey, *O. graminis* was present in asymptomatic plants, suggesting at least the occurrence of a biotrophic phase of unknown duration. As many other fungal endophytes, this species may have a latent saprobic lifestyle (Vázquez-de-Aldana et al., 2013a).

*Lachnum* is a large genus within the Hyaloscyphaceae family with more than 250 species described and distributed in a wide range of habitats and host species (Wu and Su, 2007; Nagao, 2008). Most *Lachnum* species are latent saprophytes, that may grow as endophytes in roots of perennial grasses (Sánchez-Márquez et al., 2010; Pereira et al., 2019) and form ericoid mycorrhiza coils in some Ericaceae (Walker et al., 2011). Many *Lachnum* sp. are bioactive, producing a wide range of biologically active compounds (Shan et al., 1997; Ondeyka et al., 2009; McMullin et al., 2017; Zong et al., 2017).

In addition to the core species *Omnidemptus graminis* and *Lachnum* sp., the culturable mycobiota of *B. rupestre* was characterized by a remarkable number of taxa with recognized defensive activities, such as entomopathogenic fungi (*Tolypocladium album* and *Sarocladium strictum*), (Gera Hol et al., 2007; Quesada-Moraga et al., 2014; El-Sayed et al., 2020), nematophagous fungi (*Metapochonia bulbillosa* and *Clonostachys rosea*) (Sankaranarayanan et al., 1997; Ownley et al., 2010; Manzanilla-López and Lopez-Llorca, 2017), and fungi with antifungal activity (*Fusarium circinatum*, *Penicillium ortum*, *Lachnum sp., Glarea* sp., *Trichoderma* sp., *Trichoderma koningii* and *Nemania* sp.) (Ondeyka et al., 2009; Youssar et al., 2011; Zhang et al., 2014; Mousa et al., 2015; Kornsakulkarn et al., 2017). The known defensive functions of this group of microorganisms may confer a decisive advantage to *B. rupestre*, which may use them for its own protection, as predators of its pathogens and pests, according to the bodyguard hypothesis (Elliot et al., 2000). To what extent this particular defensive assemblage plays a key role in the success and expansion of *B. rupestre* with regard to other grasses is a matter of interest that needs further research.

### Fungal Assemblages in *B. rupestre* Associated to Specific Grasslands

Low and high-diversity grasslands, generated by a distinctive disturbance regime, had similar fungal richness and diversity (Figure 4), but different endophyte assemblages (Figure 2 and PERMANOVA analysis). Differences were mostly due to the different probability of incidence of the core taxa (Figure 6). *B. rupestre* in low-diversity grasslands had a greater probability of incidence of *Omnidemptus graminis* and *Lachnum* sp. B than in high-diversity grasslands, and the two grassland types only shared 36% of the total fungal taxa identified. Considering that we sampled 120 turfs per grassland type, and that we did not reach the asymptote in the species accumulation curves (Figure 3), a more exhaustive sampling effort would be necessary to draw conclusions on a specific cohort of rare taxa per grassland type. With regard to the systemic endophyte *E. typhina*, no significant differences in the probability of incidence were found between grassland types. In consequence, the highest degree of incidence of the two core taxa, *O. graminis* (in shoots and rhizomes) and *Lachnum* sp. B (in roots and rhizomes), in low-diversity, recurrently burned grasslands was the most sound result of this part of the study.

From the spectrum of core species infecting *B. rupestre, O. graminis* was only found in shoots and rhizomes, which indicates its affinity for both tissues, which are anatomically similar (de Kroon and Knops, 1990), and different from roots (Table 3). When isolated and cultured in plates, we observed rapid mycelial growth of *O. graminis* compared to the rest of species. We hypothesize that fires destroy the aerial mycelium of *O. graminis*, together with the aboveground biomass of the plant, but the fungus remains in the rhizome reservoir. Since fire is applied in winter time in moist soils, the temperatures reached in the top soil are low (at 1 cm deep, soil temperature rise 9–10°C and no change is measured at 5 cm depth – data unpublished), and the function of rhizomes remains unaffected. Consequently, recolonization of the aboveground tissues by the rhizome mycelium of *O. graminis* can occur rapidly, paralleling the regrowth of the plant, and conferring a decisive initial advantage to this fungal species.

Regarding *Lachnum* sp. B, the increased incidence of this fungal species in frequently burnt areas paralleled a consistent decrease of *Albotricha* sp. in roots and rhizomes (Table 3). These two endophytic taxa constitute a large part of the belowground mycobiome of *B. rupestre* and belong to the same family, Hyaloscyphaceae. The close phylogeny of both genera and the polyphyly of the *Lachnum* group has been demonstrated in genetic studies (Ye and Zhuang, 2003; Hosoya et al., 2010). The pattern of increased *Lachnum* sp. and decreased *Albotricha* sp. infection in the most burned sites, may indicate a negative interaction between both species, or a contrasting response among them to the fire disturbance. The capacity of *Lachnum* sp. to cope with environmental stress, by producing sclerotia and adopting latent forms, and its growth in belowground structures, which are less affected by fire than aboveground structures, may help to explain its success. In particular, in temperate shrublands close to the region of study, ascocarps of *Lachnum pygmaeum* have been observed on charred wood and roots of *Ulex europaeus* (unpublished results). It is known that the thermal shock produced by fire may enhance fungal fecundity and activate the development of sexual structures in some pyrophilous fungi (Raudabaugh et al., 2020). Also, fungal biomass may be increased by fires, as demonstrated for the pyrophilous fungi *Morchella* sp. infecting the grass *Bromus tectorum* (Baynes et al., 2012). To what extent the particular *Lachnum* sp. B identified in the area of study is a pyrophilous fungus merits additional study.

Although the incidences of *O. graminis* and *Lachnum* sp. B are favored in such anthropized fire-prone habitats, the extent to which these endophytes confer particular advantages to its host, *B. rupestre*, is unknown. Further experimental research is needed to evaluate whether *O. graminis* and/or *Lachnum* sp. B infected plants perform better after fires than non-infected plants and whether the relationship in such a disrupted environment relies in a mutualistic relationship. In this research, the systemic *E. typhina* did not display significant differences in incidence among grasslands, and its percentage of infection was low at all sites. Previous research on *Epichloë* endophytes of grasses did not find evidence of a mutualistic relationship associated to fire (Faeth et al., 2002; Hall et al., 2014), contrary to grazing intensities that have been positively related to the abundance of vertically transmitted *Epichloë* producers of toxic metabolites (Vázquez-de-Aldana et al., 2010; Hume et al., 2020).

## Conclusion

The perennial tall-grass *B. rupestre* had a moderately diverse endophytic mycobiome consisting of a few core species and many rare species in assemblages that differed between aerial and belowground tissues. Recurrent grassland burnings, which eliminate the aerial biomass of the grass every 1–2 years, did not affect the richness and the diversity of the fungal community in *B. rupestre*, but the percentages of infection of two core taxa, *Omnidemptus graminis* and *Lachnum* sp. B, were significantly modified. The results indicate that although in frequently burnt areas the same core species of diverse grasslands subsist, *Omnidemptus graminis* and *Lachnum* sp. B are singularly benefited due to the proposed following mechanisms: (1) the capacity to survive belowground in rhizomes during the winter, and to spread rapidly to the shoots when the plant starts its spring growth (*O. graminis*), and (2) the location in belowground tissues (*Lachnum* sp. B) and the higher resistance to stress than other core root fungi, such as the related-taxon *Albotricha* sp. Following steps should address whether these two core taxa benefit the expansive success of *B. rupestre* in these antrophized, fire-prone environments, as well as to determine whether the cohort of less abundant fungi with well-defined defensive functions play a role too.

## Data Availability Statement

The datasets presented in this study can be found in online repositories. The names of the repository/repositories and accession number(s) can be found in the article/Supplementary Material.

## Author Contributions

IZ and BV introduced the training in culturable techniques for fungal identification to MD. MD and LS designed the experiments and analyzed the data. MD, LS, and LM collected the plants and isolated the fungi. MD and IZ identified the fungi. MD and RC wrote the manuscript. LS, IZ, and BV reviewed the manuscript. MD and RC got the projects that supported financially the research. All authors contributed to the article and approved the submitted version.

## Conflict of Interest

The authors declare that the research was conducted in the absence of any commercial or financial relationships that could be construed as a potential conflict of interest.

## Footnotes

1 http://meteo.navarra.es

## References

[B1] Aizpuru I. Aseginolaza C. Uribe-Echebarría P. M. Urrutia P. Zorrakin I. (1999). *Claves Ilustradas de la Flora del País Vasco y Territorios Limítrofes.* Vitoria-Gasteiz: Servicio Central de Publicaciones del Gobierno Vasco.

[B2] Alcañiz M. Outeiro L. Francos M. Úbeda X. (2018). Effects of prescribed fires on soil properties: a review. *Sci. Total Environ.* 61 944–957. 10.1016/j.scitotenv.2017.09.144 28946382

[B3] Anderson P. M. L. Hoffman M. T. (2007). The impacts of sustained heavy grazing on plant diversity and composition in lowland and upland habitats across the Kamiesberg mountain range in the Succulent Karoo, South Africa. *J. Arid Environ.* 70 686–700. 10.1016/j.jaridenv.2006.05.017

[B4] Archibald J. K. Mort M. E. Crawford D. J. Kelly J. K. (2005). Life history affects the evolution of reproductive isolation among species of *Coreopsis* (*Asteraceae*). *Evolution* 59 2362–2369. 10.1111/j.0014-3820.2005.tb00946.x 16396177

[B5] Armas-Herrera C. M. Martí C. Badía D. Ortiz-Perpiñá O. Girona-García A. Porta J. (2016). Immediate effects of prescribed burning in the central pyrenees on the amount and stability of topsoil organic matter. *CATENA* 147 238–244. 10.1016/j.catena.2016.07.016

[B6] Artz R. R. E. Reid E. Anderson I. C. Campbell C. D. Cairney J. W. G. (2009). Long term repeated prescribed burning increases evenness in the basidiomycete laccase gene pool in forest soils. *Fems Microbiol. Ecol.* 67 397–410. 10.1111/j.1574-6941.2009.00650.x 19187216

[B7] Augustine D. J. McNaughton S. J. Frank D. A. (2003). Feedbacks between soil nutrients and large herbivores in a managed savanna ecosystem. *Ecol. Appl.* 13 1325–1337. 10.1890/02-5283

[B8] Baba W. Kurowska M. Kompala-Baba A. Wilczek A. Dlugosz J. Szarejko I. (2012). Genetic diversity of populations of *Brachypodium pinnatum* (L.) P. Beauv.: expansive grass in a fragmented landscape. *Polish J. Ecol.* 60 31–40.

[B9] Bardgett R. D. Wardle D. A. (2003). Herbivore-mediated linkages between aboveground and belowground communities. *Ecology* 84 2258–2268. 10.1890/02-0274

[B10] Bates D. Mächler M. Bolker B. M. Walker S. C. (2015). Fitting linear mixed-effects models using lme4. *J. Stat. Softw.* 67 1–48. 10.18637/jss.v067.i01

[B11] Baynes M. Newcombe G. Dixon L. Castlebury L. O’Donnell K. (2012). A novel plant-fungal mutualism associated with fire. *Fungal Biol.* 116 133–144. 10.1016/j.funbio.2011.10.008 22208608

[B12] Bellgard S. E. Whelan R. J. Muston R. M. (1994). The impact of wildfire on vesicular-arbuscular mycorrhizal fungi and their potential to influence the re-establishment of post-fire plant communities. *Mycorrhiza* 4 139–146. 10.1007/BF00203532

[B13] Bråthen K. A. Jahiri X. Jusdado J. G. H. Soininen E. M. Jensen J. B. (2015). Fungal endophyte diversity in tundra grasses increases by grazing. *Fungal Ecol.* 17 41–51. 10.1016/j.funeco.2015.05.002

[B14] Brem D. Leuchtmann A. (2001). Epichloë grass endophytes increase herbivore resistance in the woodland grass *Brachypodium sylvaticum*. *Oecologia* 126 522–530. 10.1007/s004420000551 28547237

[B15] Buckland S. M. Thompson K. Hodgson J. G. Grime J. P. (2001). Grassland invasions: effects of manipulations of climate and management. *J. Appl. Ecol.* 38 301–309. 10.1046/j.1365-2664.2001.00603.x

[B16] Burgdorf R. J. Laing M. D. Morris C. D. Jamal-Ally S. F. (2014). A procedure to evaluate the efficiency of surface sterilization methods in culture-independent fungal endophyte studies. *Braz. J. Microbiol.* 45 977–983. 10.1590/S1517-83822014000300030 25477934 PMC4204985

[B17] Canals R. M. (2019). Landscape in motion: revisiting the role of key disturbances in the preservation of mountain ecosystems. *Geogr. Res. Lett.* 45 515–531. 10.18172/cig.3634

[B18] Canals R. M. Pedro J. Rupérez E. San-Emeterio L. (2014). Nutrient pulses after prescribed winter fires and preferential patterns of N uptake may contribute to the expansion of *Brachypodium pinnatum* (L.) P. Beauv. in highland grasslands. *Appl. Veg. Sci.* 17 419–428. 10.1111/avsc.12088

[B19] Canals R. M. San Emeterio L. Durán M. Múgica L. (2017). Plant-herbivory feedbacks and selective allocation of a toxic metal are behind the stability of degraded covers dominated by *Brachypodium pinnatum* in acidic soils. *Plant Soil* 415 373–386. 10.1007/s11104-016-3153-1

[B20] Canals R. M. Sebastia M. T. (2000). Soil nutrient fluxes and vegetation changes on molehills. *J. Veg. Sci.* 11 23–30. 10.2307/3236771

[B21] Changyeol L. Kim S. Li W. Bang S. Lee H. Lee H. (2017). Bioactive secondary metabolites produced by an endophytic fungus *Gaeumannomyces* sp. JS0464 from a maritime halophyte *Phragmites communis*. *J. Antibiot.* 70 737–742. 10.1038/ja.2017.39 28352106

[B22] Chen H. Boutros P. C. (2011). VennDiagram: a package for the generation of highly-customizable Venn and Euler diagrams in R. *BMC Bioinform.* 12:35. 10.1186/1471-2105-12-35 21269502 PMC3041657

[B23] Comby M. Lacoste S. Baillieul F. Profizi C. Dupont J. (2016). Spatial and temporal variation of cultivable communities of co-occurring endophytes and pathogens in wheat. *Front. Microbiol.* 7:403. 10.3389/fmicb.2016.00403 27065969 PMC4814462

[B24] Constantin M. E. Vlieger B. V. Takken F. I. Rep M. (2020). Diminished pathogen and enhanced endophyte colonization upon coinoculation of endophytic and pathogenic *Fusarium* strains. *Microorganisms* 8:544. 10.3390/microorganisms8040544 32283705 PMC7232452

[B25] Dawson L. A. Mayes R. W. Elston D. A. Smart T. S. (2000). Root hydrocarbons as potential markers for determining species composition. *Plant Cell Environ.* 23 743–750. 10.1046/j.1365-3040.2000.00592.x

[B26] de Kroon H. Knops J. (1990). Habitat exploration through morphological plasticity in two chalk grassland perennials. *Oikos* 59 39–49. 10.2307/3545120

[B27] Dufrêne M. Legendre P. (1997). Species assemblages and indicator species: the need for a flexible asymmetrical approach. *Ecol. Monogr.* 67 345–366. 10.2307/2963459

[B28] Durán M. Canals R. M. Sáez J. L. Ferrer V. Lera-López F. (2020). Disruption of traditional land use regimes causes an economic loss of provisioning services in high-mountain grasslands. *Ecosyst. Servic.* 46:101200. 10.1016/j.ecoser.2020.101200

[B29] Egidi E. McMullan-Fisher S. Morgan J. W. May T. Zeeman B. Franks A. E. (2016). Fire regime, not time-since-fire, affects soil fungal community diversity and composition in temperate grasslands. *FEMS Microbiol. Lett.* 363 1–11. 10.1093/femsle/fnw196 27528692

[B30] Eldridge D. J. Delgado-Baquerizo M. (2018). Functional groups of soil fungi decline under grazing. *Plant Soil* 426 51–60. 10.1007/s11104-018-3617-6

[B31] Elliot S. L. Sabelis M. W. Janssen A. van der Geest L. P. S. Beerling E. A. M. Fransen J. (2000). Can plants use entomopathogens as bodyguards? *Ecol. Lett.* 3 228–235. 10.1046/j.1461-0248.2000.00137.x

[B32] El-Sayed A. S. A. Moustafa A. H. Hussein H. A. El-Sheikh A. A. El-Shafey S. N. Fathy N. A. M. (2020). Potential insecticidial activity of *Sarocladium strictum*, an endophyte of *Cynanchum acutum*, against *Spodoptera littorales*, a polyphagous insect pest. *Biocatal. Agric. Biotechnol.* 24:101524. 10.1016/j.bcab.2020.101524

[B33] Eom A. H. Hartnett D. C. Wilson G. W. T. Figge D. A. H. (1999). The effect of fire, mowing and fertilizer amendment on arbuscular mycorrhizas in tallgrass prairie. *Am. Midl. Nat.* 142 55–70.

[B34] Faeth S. H. Haase S. M. Sackett S. S. Sullivan T. J. Remington R. K. Hamilton C. E. (2002). Does fire maintain symbiotic, fungal endophyte infections in native grasses? *Symbiosis* 32 211–228.

[B35] Ferrer V. Canals R. M. (2008). *Proyecto de Ordenación de los Recursos Pascícolas Forestales del Monte Aezkoa n° 1 del CUP.* Pamplona: Consultoría Belardi & Universidad Pública de Navarra.

[B36] Frank D. A. (2005). The interactive effects of grazing ungulates and aboveground production on grassland diversity. *Oecologia* 143 629–634. 10.1007/s00442-005-0019-2 15800752

[B37] Gera Hol W. H. de la Peña E. Moens M. Cook R. (2007). Interaction between a fungal endophyte and root herbivores of *Ammophila arenaria*. *Basic Appl. Ecol.* 8 500–509. 10.1016/j.baae.2006.09.013

[B38] Giauque H. Hawkes C. V. (2016). Historical and current climate drive spatial and temporal patterns in fungal endophyte diversity. *Fungal Ecol.* 20 108–114. 10.1016/j.funeco.2015.12.005

[B39] Glynou K. Ali T. Buch A.-K. Kia S. H. Ploch S. Xia X. (2016). The local environment determines the assembly of root endophytic fungi at a continental scale. *Environ. Microbiol.* 18 2418–2434. 10.1111/1462-2920.13112 26530450

[B40] Hall S. L. McCulley R. L. Barney R. J. Phillips T. D. (2014). Does fungal endophyte infection improve tall fescue’s growth response to fire and water limitation? *PLoS One* 9:e0086904. 10.1371/journal.pone.0086904 24497994 PMC3908949

[B41] Hamilton E. W. Frank D. A. (2001). Can plants stimulate soil microbes and their own nutrient supply? Evidence from a grazing tolerant grass. *Ecology* 82 2397–2402.

[B42] Harrison J. G. Griffin E. A. (2020). The diversity and distribution of endophytes across biomes, plant phylogeny and host tissues: how far have we come and where do we go from here? *Environ. Microbiol.* 22 2107–2123. 10.1111/1462-2920.14968 32115818 PMC7679042

[B43] Hernández-Restrepo M. Bezerra J. D. P. Tan Y. P. Wiederhold N. Crous P. W. Guarro J. (2019). Re-evaluation of *Mycoleptodiscus* species and morphologically similar fungi. *Persoonia* 42 205–227. 10.3767/persoonia.2019.42.08 31551619 PMC6712544

[B44] Higgins K. L. Arnold A. E. Coley P. D. Kursar T. A. (2014). Communities of fungal endophytes in tropical forest grasses: highly diverse host- and habitat generalists characterized by strong spatial structure. *Fungal Ecol.* 8 1–11. 10.1016/j.funeco.2013.12.005

[B45] Hiiesalu I. Bahram M. Tedersoo L. (2017). Plant species richness and productivity determine the diversity of soil fungal guilds in temperate coniferous forest and bog habitats. *Mol. Ecol.* 26 4846–4858. 10.1111/mec.14246 28734072

[B46] Hill N. S. Stringer W. C. Rottinghaus G. E. Belesky D. P. Parrott W. A. Pope D. D. (1989). Influence of endophyte and water regime upon tall fescue accessions. I. Growth characteristics. *Ann. Bot.* 63 495–503. 10.1093/oxfordjournals.aob.a087775

[B47] Holub P. Tuma I. Fiala K. (2012). The effect of nitrogen addition on biomass production and competition in three expansive tall grasses. *Environ. Pollut.* 170 211–216. 10.1016/j.envpol.2012.07.007 22835500

[B48] Hosoya T. Sasagawa R. Hosaka K. Gi-Ho S. Hirayama Y. Yamaguchi K. (2010). Molecular phylogenetic studies of *Lachnum* and its allies based on the Japanese material. *Mycoscience* 51 170–181. 10.1007/s10267-009-0023-1

[B49] Hothorn T. Van De Wiel M. A. Hornik K. Zeileis A. (2008). Implementing a class of permutation tests: the coin package. *J. Stat. Softw.* 28 1–23. 10.18637/jss.v028.i0827774042

[B50] Huang Y. L. Devan M. M. N. U’Ren J. M. Furr S. H. Arnold A. E. (2016). Pervasive effects of wildfire on foliar endophyte communities in montane forest trees. *Microb. Ecol.* 71 452–468. 10.1007/s00248-015-0664-x 26370111 PMC4729612

[B51] Huang Y. L. Niu B. F. Gao Y. Fu L. M. Li W. Z. (2010). CD-HIT Suite: a web server for clustering and comparing biological sequences. *Bioinformatics* 26 680–682. 10.1093/bioinformatics/btq003 20053844 PMC2828112

[B52] Hume D. E. Stewart A. V. Simpson W. R. Johnson R. D. (2020). *Epichloë* fungal endophytes play a fundamental role in New Zealand grasslands. *J. Roy. Soc. N. Z.* 20 279–298. 10.1080/03036758.2020.1726415

[B53] Hyde K. D. Soytong K. (2008). The fungal endophyte dilemma. *Fungal Divers.* 33 133–173.

[B54] Illana A. Rodriguez-Romero J. Sesma A. (2013). “major plant pathogens of the magnaporthaceae family genomics of soil- and plant-associated fungi,” in *Genomics of Soil- and Plant-Associated Fungi*, eds Horwitz B. A. Mukherjee P. K. Mukherjee M. Kubicek C. P. (Berlin: Springer Berlin Heidelberg), 45–88. 10.1007/978-3-642-39339-6_4

[B55] Jing Z. Cheng J. Su J. Bai Y. Jin J. (2014). Changes in plant community composition and soil properties under 3-decade grazing exclusion in semiarid grassland. *Ecol. Eng.* 64 171–178. 10.1016/j.ecoleng.2013.12.023

[B56] Kankanala P. Czymmek K. Valent B. (2007). Roles for rice membrane dynamics and plasmodesmata during biotrophic invasion by the blast fungus. *Plant Cell* 19 706–724. 10.1105/tpc.106.046300 17322409 PMC1867340

[B57] Kivlin S. N. Kazenel M. R. Lynn J. S. Lee Taylor D. Rudgers J. A. (2019). Plant identity influences foliar fungal symbionts more than elevation in the colorado rocky mountains. *Microb. Ecol.* 78 688–698. 10.1007/s00248-019-01336-4 30715579

[B58] Köhler B. Gigon A. Edwards P. J. Krüsi B. Langenauer R. Lüscher A. (2005). Changes in the species composition and conservation value of limestone grasslands in Northern Switzerland after 22 years of contrasting managements. *Perspect. Plant Ecol. Evol. Syst.* 7 51–67. 10.1016/j.ppees.2004.11.003

[B59] Koide R. T. Ricks K. D. Davis E. R. (2017). Climate and dispersal influence the structure of leaf fungal endophyte communities of *Quercus gambelii* in the eastern Great Basin. USA. *Fungal Ecol.* 30 19–28. 10.1016/j.funeco.2017.08.002

[B60] Komac B. Alados C. L. Bueno C. G. Gomez D. (2013). Spatial patterns of species distributions in grazed subalpine grasslands. *Plant Ecol.* 212 519–529.

[B61] Kornsakulkarn J. Saepua S. Suvannakad R. Supothina S. Boonyuen N. Isaka M. (2017). Cytotoxic tropolones from the fungus *Nemania* sp. BCC30850. *Tetrahedron* 73 3505–3512. 10.1016/j.tet.2017.05.030

[B62] Kotilínek M. Hiiesalu I. Košnar J. Šmilauerová M. Šmilauer P. Altman J. (2017). Fungal root symbionts of high-altitude vascular plants in the Himalayas. *Sci. Rep.* 7 1–14. 10.1038/s41598-017-06938-x 28747779 PMC5529584

[B63] Lasanta-Martínez T. Vicente-Serrano S. M. Cuadrat-Prats J. M. (2005). Mountain mediterranean landscape evolution caused by the abandonment of traditional primary activities: a study of the spanish central pyrenees. *Appl. Geogr.* 25 47–65. 10.1016/j.apgeog.2004.11.001

[B64] Lau M. K. Arnold A. E. Johnson N. C. (2013). Factors influencing communities of foliar fungal endophytes in riparian woody plants. *Fungal Ecol.* 6 365–378. 10.1016/j.funeco.2013.06.003

[B65] Leinonen P. H. Helander M. Vázquez-de-Aldana B. R. Zabalgogeazcoa I. Saikkonen K. (2019). Local adaptation in natural European host grass populations with asymmetric symbiosis. *PLoS One* 14:e0215510. 10.1371/journal.pone.0215510 30995278 PMC6469795

[B66] Leuchtmann A. Bacon C. W. Schardl L. White J. F. Tadych M. (2014). Nomenclatural realignment of *Neotyphodium* species with genus *Epichloë*. *Mycologia* 106 202–215. 10.3852/13-251 24459125

[B67] Leuchtmann A. Schardl C. L. (1998). Mating compatibility and phylogenetic relationships among two new species of *Epichloë* and other congeneric European species. *Mycol. Res.* 102 1169–1182. 10.1017/S0953756298006236

[B68] Li H. Y. Shen M. Zhou Z. P. Li T. Wei Y. L. Lin L. B. (2012). Diversity and cold adaptation of endophytic fungi from five dominant plant species collected from the Baima Snow Mountain. Southwest China. *Fungal Divers.* 54 79–86. 10.1007/s13225-012-0153-1

[B69] Li W. Godzik A. (2006). Cd-hit: a fast program for cluestering and comparing large sets of protein or nucleotide sequences. *Bioinformatics Appl. Note* 22 1658–1659. 10.1093/bioinformatics/btl158 16731699

[B70] Liu C. Wang L. Song X. Chang Q. Frank D. A. Wang D. (2017). Towards a mechanistic understanding of the effect that different species of large grazers have on grassland soil N availability. *J. Ecol.* 106 357–366. 10.1111/1365-2745.12809

[B71] Malinowski D. Belesky P. (2000). Adaptations of endophyte-infected cool-season grasses to environmental stresses: mechanisms of drought and mineral stress tolerance. *Crop Sci.* 40 923–940. 10.2135/cropsci2004.0523

[B72] Manzanilla-López R. Lopez-Llorca L. V. (2017). “Perspectives in sustainable nematode management through *Pochonia chlamydosporia* applications for root and rhizosphere health,” in *Sustainability in Plant Crop Protection*, eds Peshin R. Dhawan A. (Berlin: Springer), 10.1007/978-3-319-99768-1

[B73] Mataix-Solera J. Navarro-Pedreno J. Guerrero C. Gomez I. Marco B. Mataix J. (2002). “Effects of an experimental fire on soil microbial populations in a Mediterranean environment,” in *Man and Soil at the Third Millennium. Proceedings International Congress of the European Society for Soil Conservation*, Vol. 2 eds Rubio J. L. Morgan R. P. C. Asins S. Andreu V. (Valencia), 1607–1613. Geoforma Ediciones.

[B74] McMullin D. R. Green B. D. Prince N. C. Tanney J. B. Miller D. J. (2017). Natural products of *Picea* endophytes from the acadian forest. *J. Nat. Prod.* 80 1475–1483. 10.1021/acs.jnatprod.6b01157 28398744

[B75] Meijer G. Leuchtmann A. (1999). Multistrain infections of the grass *Brachypodium sylvaticum* by its fungal endophyte *Epichloë sylvatica*. *New Phytol.* 141 355–368. 10.1046/j.1469-8137.1999.00332.x 33862916

[B76] Milchunas D. G. Sala O. E. Lauenroth W. K. (1988). A generalized model of the effects of grazing by large herbivores on grassland community structure. *Am. Nat.* 132 87–106.

[B77] Mousa W. K. Schwan A. Davidson J. Strange P. Liu H. Zhou T. (2015). An endophytic fungus isolated from finger millet (*Eleusine coracana*) produces anti-fungal natural products. *Front. Microbiol.* 6:1157. 10.3389/fmicb.2015.01157 26539183 PMC4612689

[B78] Múgica L. Canals R. M. San Emeterio L. (2018). Changes in soil nitrogen dynamics caused by prescribed fires in dense gorse lands in SW Pyrenees. *Sci. Total Environ.* 639 175–185. 10.1016/j.scitotenv.2018.05.139 29783117

[B79] Nagao H. (2008). Discomycetes on decayed tree fern. *Lachnum lanariceps* and *Lachnum oncospermatum* new to Japan. *Mycoscience* 49 403–406. 10.1007/S10267-008-0438-0

[B80] Nguyen N. H. Song Z. Bates S. T. Branco S. Tedersoo L. Menke J. (2016). FUNGuild: An open annotation tool for parsing fungal community datasets by ecological guild. *Fungal Ecol.* 20, 241–248. 10.1016/j.funeco.2015.06.006

[B81] Ofek-Lalzar M. Gur Y. Ben-Moshe S. Sharon O. Kosman E. Mochli E. (2016). Diversity of fungal endophytes in recent and ancient wheat ancestors *Triticum dicoccoides* and *Aegilops sharonensis*. *FEMS Microbiolo. Ecol.* 92:fiw152. 10.1093/femsec/fiw152 27402714

[B82] Oksanen J. Blanchet F. G. Friendly M. Kindt R. Legendre P. McGlinn D. (2017). *Vegan: Community Ecology Package. R Package Version 2.4-3.* Available online at: https://CRAN.R-project.org/package=vegan (accessed April 10, 2019).

[B83] Ondeyka J. Harris G. Zink D. Basilio A. Vicente F. Bills G. (2009). Isolation, structure elucidation, and biological activity of virgineone from *Lachnum virgineum* using the genome-wide *Candida albicans* fitness test. *J. Nat. Prod.* 72 136–141. 10.1021/np800511r 19115836

[B84] Ownley B. H. Gwinn K. D. Vega F. E. (2010). Endophytic fungal entomopathogens with activity against plant pathogens: ecology and evolution. *BioControl* 55 113–128. 10.1007/s10526-009-9241-x

[B85] Pauchard A. Kueffer C. Dietz H. Daehler C. C. Alexander J. Edwards P. J. (2009). Ain’t no mountain high enough: plant invasions reaching new elevations. *Front. Ecol. Environ.* 7:479–486. 10.1890/080072

[B86] Pellegrini A. F. A. McLauchlan K. K. Hobbie S. E. Mack M. C. Marcotte A. L. Nelson D. M. (2020). Frequent burning causes large losses of carbon from deep soil layers in a temperate savanna. *J. Ecol.* 108 1426–1441. 10.1111/1365-2745.13351

[B87] Pereira E. Vázquez de Aldana B. R. San Emeterio L. Zabalgogeazcoa Í (2019). A survey of culturable fungal endophytes from *Festuca rubra* subsp. *pruinosa*, a grass from marine cliffs, reveals a core microbiomes. *Front. Microbiol.* 9:3321. 10.3389/fmicb.2018.03321 30700985 PMC6343541

[B88] Poosakkannu A. Nissinen R. Kytöviita M.-M. (2014). Culturable endophytic microbial communities in the circumpolar grass, *Deschampsia flexuosa* in a sub-Arctic inland primary succession are habitat and growth stage specific. *Environ. Micribiol. Rep.* 7 111–122. 10.1111/1758-2229.12195 25721603

[B89] Quesada-Moraga E. Herrero N. Zabalgogeazcoa Í (2014). “Entomopathogenic and nematophagous fungal endophytes,” in *Advances in Endophytic Research*, eds Verma V. Gange A. (New Delhi: Springer), 10.1007/978-81-322-1575-2_4

[B90] Ranelli L. B. Hendricks W. Q. Lynn J. S. Kivlin S. N. Rudgers J. A. (2015). Biotic and abiotic predictors of fungal colonization in grasses of the Colorado Rockies. *Divers. Distrib.* 21 962–976. 10.1111/ddi.12310

[B91] Raudabaugh D. B. Matheny P. B. Hughes K. W. Iturriaga T. Sargent M. Miller A. N. (2020). Where are they hiding? Testing the body snatchers hypothesis in pyrophilous fungi. *Fungal Ecol.* 43:100870. 10.1016/j.funeco.2019.100870

[B92] Roberts D. W. (2019). *Labdsv: Ordination and Multivariate Analysis for Ecology. R Package Version 1.8-0.*

[B93] Rudgers J. A. Kivlin S. N. Whitney K. D. Price M. V. Waser N. M. Harte J. (2014). Responses of high-altitude graminoids and soil fungi to 20 years of experimental warming. *Ecology* 95 1918–1928. 10.1890/13-1454.1 25163124

[B94] Saikkonen K. Ion D. Gyllenberg M. (2002). The persistence of vertically transmitted fungi in grass metapopulations. *Proc. R. Soc. Lond. B* 269 1397–1403. 10.1098/rspb.2002.2006 12079664 PMC1691040

[B95] Saikkonen K. Young C. A. Helander M. Schardl C. L. (2016). Endophytic *Epichloë* species and their grass hosts: from evolution to applications. *Plant Mol. Biol.* 90 665–675. 10.1007/s11103-015-0399-6 26542393 PMC4819788

[B96] San Emeterio L. Múgica L. Ugarte M. D. Goicoa T. Canals R. M. (2016). Sustainability of traditional pastoral fires in highlands under global change: effects on soil function and nutrient cycling. *Agric. Ecosyst. Environ.* 235 155–163. 10.1016/j.agee.2016.10.009

[B97] Sánchez-Márquez S. Bills G. F. Acuña L. D. Zabalgogeazcoa I. (2010). Endophytic mycobiota of leaves and roots of the grass *Holcus lanatus*. *Fungal Divers.* 41 115–123.

[B98] Sánchez-Márquez S. Bills G. F. Zabalgogeazcoa I. (2008). Diversity and structure of the fungal endophytic assemblages from two sympatric coastal grasses. *Fungal Divers.* 33 87–100.

[B99] Sankaranarayanan C. Hussaini S. S. Kumar P. S. Prasad R. D. (1997). Nematicidal effect of fungal filtrates against root-knot nematodes. *J. Biol. Control* 11 37–41. 10.18311/jbc/1997/7575

[B100] Schardl C. L. Balestrini R. Florea S. Zhang D. Scott B. (2009). “*Epichloë* endophytes: clavicipitaceous symbionts of grasses,” in *Plant Relationships. The Mycota (A Comprehensive Treatise on Fungi as Experimental Systems for Basic and Applied Research)*, Vol. 5 ed. Deising H. B. (Berlin: Springer Berlin Heidelberg), 275–306. 10.1007/978-3-540-87407-2_15

[B101] Schippmann U. (1991). Revision der europäischen arten der gattung *Brachypodium* palisot de beauvois (*Poaceae*). *Boissiera* 45 1–250.

[B102] Schippmann U. Jarvis C. E. (1988). Typification of three Linnean names associated with the genus *Brachypodium* (*Poaceae*). *Taxon* 37 158–164. 10.2307/1220950

[B103] Schirrmann M. K. Zoller S. Fior S. Leuchtmann A. (2015). Genetic evidence for reproductive isolation among sympatric *Epichloë* endophytes as inferred from newly developed microsatellite markers. *Microb. Ecol.* 70 51–60. 10.1007/s00248-014-0556-5 25542204

[B104] Schoch C. L. Robbertse B. Robert V. Vu, D. Cardinali G. Iriny L. (2014). Finding needles in haystacks: linking scientific names, reference specimens and molecular data for Fungi. *Database (Oxford)* 2014:bau061. 10.1093/database/bau061 24980130 PMC4075928

[B105] Schulz B. Boyle C. (2005). The endophytic continuum. *Mycol. Res.* 109 661–686. 10.1017/S095375620500273X 16080390

[B106] Semenova-Nelsen T. A. Platt W. J. Patterson T. R. Huffman J. Sikes B. A. (2019). Frequent fire reorganizes fungal communities and slows decomposition across a heterogeneous pine savanna landscape. *New Phytol.* 224 916–927. 10.1111/nph.16096 31396966

[B107] Shan R. Stadler M. Anke H. Sterner O. (1997). Naphthalenone and phthalide metabolites from *Lachnum papyraceum*. *J. Nat. Prod.* 60 804–805. 10.1021/np970145s

[B108] Shaw E. A. Denef K. de Tomasel C. Cotrufo M. F. Wall D. H. (2016). Fire affects root decomposition, soil food web structure, and carbon flow in tallgrass prairie. *SOIL* 2 199–210. 10.5194/soil-2-199-2016

[B109] Slaughter L. C. Nelson J. A. Carlisle E. Bourguignon M. Dinkind R. D. Phillips T. D. (2018). Climate change and *Epichloë coenophiala* association modify belowground fungal symbioses of tall fescue host. *Fungal Ecol.* 31 37–46. 10.1007/s00248-016-0828-3 27502203

[B110] Soong J. L. Cotrufo M. F. (2015). Annual burning of a tallgrass prairie inhibits C and N cycling in soil, increasing recalcitrant pyrogenic organic matter storage while reducing N availability. *Glob. Change Biol.* 21 2321–2333. 10.1111/gcb.12832 25487951

[B111] Sun X. Kosman E. Sharon O. Ezrati S. Sharon A. (2020). Significant host- and environment-dependent differentiation among highly sporadic fungal endophyte communities in cereal crops-related wild grasses. *Environ. Microbiol.* 22 3357–3374. 10.1111/1462-2920.15107 32483901

[B112] Talbot N. J. McCafferty H. R. K. Ma M. Moore K. Hamer J. E. (1997). Nitrogen starvation of the rice blast fungus Magnaporthe grisea may act as an environmental cue for disease symptom expression. *Physiol. Mol. Plant Pathol.* 50 179–195. 10.1006/pmpp.1997.0081

[B113] Tardella F. M. Malatesta L. Goia I. G. Catorci A. (2018). Effects of long-term mowing on coenological composition and recovery routes of a *Brachypodium rupestre*-invaded community: insight into the restoration of sub-Mediterranean productive grasslands. *Rend. Lincei Sci. Fis. Nat.* 29 329–341.

[B114] Taylor J. W. Jacobson D. J. Kroken S. Kasuga T. Geiser D. M. Hibbett D. S. (2000). Phylogenetic species recognition and species concepts in fungi. *Fungal Genet. Biol.* 31 21–32. 11118132 10.1006/fgbi.2000.1228

[B115] Uys R. G. Bond W. J. Everson T. M. (2004). The effect of different fire regimes on plant diversity in southern African grasslands. *Biol. Conserv.* 118 489–499. 10.1016/j.biocon.2003.09.024

[B116] Vandenkoornhuyse P. Quaiser A. Duhamel M. Le Van A. Dufresne A. (2015). The importance of the microbiome of the plant holobiont. *New Phytol.* 206 1196–1206. 10.1111/nph.13312 25655016

[B117] Vannier N. Bittebiere A. K. Mony C. Vandenkoornhuyse P. (2020). Root endophytic fungi impact host plant biomass and respond to plant composition at varying spatio-temporal scales. *Fungal Ecol.* 44:100907. 10.1016/j.funeco.2019.100907

[B118] Vázquez-de-Aldana B. R. Bills G. Zabalgogeazcoa I. (2013a). Are endophytes an important link between airborne spores and allergen exposure? *Fungal Divers.* 60 33–42. 10.1007/s13225-013-0223-z

[B119] Vázquez-de-Aldana B. R. García-Ciudad A. García-Criado B. Vicente-Tavera S. Zabalgogeazcoa I. (2013b). Fungal endophyte (*Epichloë festucae*) alters the nutrient content of *Festuca rubra* regardless of water availability. *PLoS One* 8:e84539. 10.1371/journal.pone.0084539 24367672 PMC3867530

[B120] Vázquez-de-Aldana B. R. Zabalgogeazcoa I. Rubio de Casas R. García-Ciudad A. García-Criado B. (2010). Relationships between the genetic distance of *Epichloë festucae* isolates and the ergovaline and peramine contents of their *Festuca rubra* hosts. *Ann. Appl. Biol.* 156 51–61. 10.1111/j.1744-7348.2009.00360.x

[B121] Walker J. F. Aldrich-Wolfe L. Riffel A. Barbare H. Simpson N. B. Trowbridge J. (2011). Diverse Helotiales associated with the roots of three species of Arctic Ericaceae provide no evidence for host specificity. *New Phytol.* 191 515–527. 10.1111/j.1469-8137.2011.03703.x 21463329

[B122] Wang Y. Liu L. Yang J. Duan Y. Luo Y. Taherzadeh M. J. (2020). The diversity of microbial community and function varied in response to different agricultural residues composting. *Sci. Total Environ.* 715:136983. 10.1016/j.scitotenv.2020.136983 32041001

[B123] White T. J. Bruns T. Lee S. Taylor J. (1990). “Amplificacition and direct sequencing of fungal ribosomal RNA genes for phylogenetics,” in *PCR Protocols: A Guide to Methods and Applications*, ed. Innis M. A. (San Diego, CA: Academic Press), 315–322.

[B124] Wu M. Su Y. (2007). *Lachnum alnifolium*,a foliicolous discomycetes new to Taiwan. *Fung. Sci.* 22 85–89.

[B125] Ye M. Zhuang W.-Y. (2003). New taxa of Lachnum (Helotiales. Hyaloscyphaceae) from temperate China. *Nova Hedwigia* 76 443–450. 10.1127/0029-5035/2003/0076-0443

[B126] Youssar L. Grüning B. A. Erxleben A. Günther S. Hüttel W. (2011). Genome sequence of the fungus *Glarea lozoyensis*: the first genome sequence of a species from the Helotiaceae family. *Eukaryotic Cell* 11:250. 10.1128/EC.00142-12 PMC3272893 22302591

[B127] Yuan Z. Lin F. Zhang C. Kubicek C. P. (2010). A new species of *Harpophora* (Magnaporthaceae) recovered from healthy wild rice (*Oryza granulata*) roots, representing a novel member of a beneficial dark septate endophyte. *FEMS Microbiol. Lett.* 307 94–101. 10.1111/j.1574-6968.2010.01963.x 20402786

[B128] Zabalgogeazcoa I. Gundel P. Helander M. Saikkonen K. (2013). Non-systemic fungal endophytes in *Festuca rubra* plants infected by *Epichloë festucae* in subarctic habitats. *Fungal Divers.* 60 25–32.

[B129] Zaurov D. E. Bonos S. Murphy J. A. Richardson M. Belanger F. C. (2001). Endophyte infection can contribute to aluminum tolerance in fine fescues. *Crop Sci.* 41 1981–1984. 10.2135/cropsci2001.1981

[B130] Zhang Q. Zhang J. Yang L. Zhang L. Jiang D. Chen W. (2014). Diversity and biocontrol potential of endophytic fungi in *Brassica napus*. *Biol. Control* 72 98–108. 10.1016/j.biocontrol.2014.02.018

[B131] Zong S. Ji J. Li J. Yang Q. H. Ye M. (2017). Physicochemical properties and anticoagulant activity of polyphenols derived from *Lachnum singerianum*. *J. Food Drug Anal.* 25 837–844. 10.1016/j.jfda.2016.08.011 28987360 PMC9328885

