# Supplementary material for: Disruption of Traditional Grazing and Fire Regimes Shape the Fungal Endophyte Assemblages of the Tall-Grass Brachypodium rupestre
Source: Front Microbiol. 2021 Jun 11;12:679729. doi: 10.3389/fmicb.2021.679729 (PMC8226146; doi:10.3389/fmicb.2021.679729)
Supplement: Supplementary file 1 [file Table_1.DOCX]

Supplementary Material

**Supplementary material, table S1:** The proposed identification of the isolated taxa based on the information provided by the matches in the databases.

| **GenBank accession number** | **Greatest sequence identity (%)** | **Proposed taxon** | **Guild** | **Supplementary references** |
| --- | --- | --- | --- | --- |
| MW528548 | *Acremonium furcatum* (98.07) | *Acremonium* sp. | Animal Pathogen Endophyte Fungal Parasite Plant Pathogen Wood Saprotroph | Tian *et al.,* 2017  An *et al.,* 1993 |
| MW528549 | *Albotricha* spp. (98.46) | *Albotricha* sp. | Undefined Saprotroph | Wu M-L., 2003 |
| MW528550 | *Alfaria thymi* (100) | *Alfaria thymi* | Undefined | Lombard *et al.,* 2016 |
| MW528551 | *Funiliomyces biseptatus* (92.31) | Amphisphaeriaceae sp. | Undefined Saprotroph | Kang *et al.,* 1999 |
| MW528552 | *Bullanockia australis* (99.59) | *Bullanockia australis* | Undefined Saprotroph | Crous *et al.,* 2016 |
| MW528553 | *Cadophora obovata* (99.47) | *Cadophora* sp. | Endophyte | Newsham K.K., 2010 |
| MW528554 | *Chaetosphaeria ciliata* (98) | *Chaetosphaeria* sp. | Undefined Saprotroph | Réblová *et al.,* 1999 |
| MW528555 | Chaetosphaeriales (100) | Chaetosphaeriales sp. | Undefined | Maharachchikumbura *et al*., 2016 |
| MW528556 | *Clonostachys rosea* (100) | *Clonostachys rosea* | Plant Saprotroph Wood Saprotroph  Animal Pathogen | Rodríguez *et al.,* 2011  Lahlali and Peng*,* 2014  Fournier *et al.,* 2020 |
| MW528557 | Cucurbitariaceae spp. (94.76) | Cucurbitariaceae sp. | Plant Pathogen Wood Saprotroph | Doilom *et al.,* 2013 |
| MW528558 | *Dictyochaeta lithocarpi* (98) | *Dictyochaeta* sp. | Undefined Saprotroph | Réblová *et al.,* 1999 |
| MW528559 | *Dinemasporium morbidum* (100) | *Dinemasporium morbidum* | Undefined Saprotroph | Krohn *et al.,* 2008  Crous *et al.,* 2012 |
| MW528560 | *Epichloë typhina* (100) | *Epichloë typhina* | Endophyte Plant pathogen | White J.F., 1988 Zabalgogeazcoa *et al.,* 2007  Clay and Schardl, 2002 |
| MW528561 | *Fusarium circinatum* (100) | *Fusarium circinatum* | Animal Pathogen Endophyte Lichen Parasite Plant Pathogen Soil Saprotroph Wood Saprotroph | Hanada *et. al.,* 2010  Martínez-Álvarez *et al.,* 2016 |
| MW528562 | *Gaeumannomycella cariciola* (96.59) | *Gaeumannomycella* sp. | Plant Pathogen | Hernández-Restrepo *et al.,* 2016a |
| MW528563 | *Glarea lozoyensis* (96.59) | *Glarea* sp. | Undefined Saprotroph | Youssar *et. al.,* 2011 |
| MW528564 | Hypocreales spp. (94.54) | Hypocreales sp. |  |  |
| MW528565 | *Ilyonectria robusta* (100) | *Ilyonectria robusta* | Plant Pathogen | Martínez-Diz *et al.,* 2018  Liu *et al.,* 2019 |
| MW528566 | *Lachnum pygmaeum* (99.01) | *Lachnum* sp. A | Undefined Saprotroph | Pereira *et al.,* 2019  McMullin *et al.,* 2017  Xu *et al.,* 2017 Hou et al., 2019 |
| MW528567 | *Lachnum* spp. (99.04) | *Lachnum* sp. B | Undefined Saprotroph |  |
| MW528568 | *Metapochonia bulbillosa* (99.16) | *Metapochonia bulbillosa* | Animal Pathogen | Manzanilla-López and Lopez-Llorca, 2017 |
| MW528569 | *Metarhizium carneum* (98.7) | *Metarhizium carneum* | Animal Pathogen | Lomer *et al.,* 2001  Inyang *et al.,* 1998 |
| MW528570 | *Microdochium bolleyi* (99.57) | *Microdochium bolleyi* | Plant Pathogen | Ernst *et al.,* 2011  Shadmani *et al. ,* 2020 Zhang *et al*., 2008 |
| MW528571 | *Microdochium neoqueenslandicum* (99.77) | *Microdochium neoqueenslandicum* | Plant Pathogen | Hernández-Restrepo *et al*., 2016b |
| MW528572 | *Mollisia* spp. (97.88) | *Mollisia* sp. | Endophyte Plant Pathogen | Tanney and Seifert, 2020  Fan *et al., 2016* |
| MW528573 | *Loramyces* spp. (99.40) | Mollisiaceae sp. | Endophyte Plant Pathogen | Tanney and Seifert, 2020 |
| MW528574 | *Mycena fulgoris* (96) | *Mycena* sp. | Leaf Saprotroph Plant Pathogen Undefined Saprotroph Wood Saprotroph | Tejesvi *et al.,* 2010  Ogura-Tsujite *et. al.,* 2009 |
| MW528575 | *Myrmecridium phragmitis* (95.89) | *Myrmecridium* sp. | Endophyte | Jie *et al.,* 2013 |
| MW528576 | *Myrothecium* spp. (100) | *Myrothecium* sp. | Undefined Saprotroph | Clarke *et al.,* 2007  Hoagland *et al.,* 2007  Bezerra *et al.,* 2015 |
| MW528577 | *Nemania* spp. (99.79) | *Nemania* sp. | Undefined Saprotroph | Kornsakulkarn *et al.,* 2017 |
| MW528578 | *Omnidemptus graminis* (99.54) | *Omnidemptus graminis* | Plant Pathogen | Hernández-Restrepo *et al.,* 2019  Ortega *et al.,* 2013 |
| MW528579 | *Ophiosphaerella korrae* (100) | *Ophiosphaerella* sp. | Plant Pathogen | Flores *et al.,* 2015 |
| MW528580 | *Parasola crataegi* (96.27) | *Parasola* sp. | Undefined Saprotroph | Szarkándi *et al.,* 2017 |
| MW528581 | *Penicillium ortum* (100) | *Penicillium ortum* | Wood Saprotroph | Dastogeer *et al.,* 2018  Mousa *et al.,* 2015  Visagie *et al.,* 2015 |
| MW528582 | *Vararia calami* (84.06) | Peniophoraceae sp. | Plant Pathogen Wood Saprotroph | Miller *et al*., 2006 |
| MW528583 | *Periconia* spp. (99.8) | *Periconia* sp. | Endophyte Plant Pathogen Wood Saprotroph | *Liu et al. ,* 2017 |
| MW528584 | *Petrakia* spp. (99.19) | *Petrakia* sp. | Undefined Saprotroph | Jaklitsch and Voglmayr*,* 2017 Gross *et al. ,* 2017 |
| MW528585 | *Pezicula rhizophila* (100) | *Pezicula rhizophila* | Undefined Saprotroph | Chen *et al.,* 2016  Lynch *et al.,* 2013 |
| MW528586 | Pleosporales spp. (99.68) | Pleosporales sp. |  |  |
| MW528587 | *Pyrenochaetopsis leptospora* (100) | *Pyrenochaetopsis leptospora* | Endophyte Lichen Parasite Undefined Saprotroph | Doilom *et al.,* 2013  Chi *et al.,* 2019 |
| MW528588 | *Sarocladium strictum* (98.96) | *Sarocladium strictum* | Undefined Saprotroph  Animal pathogen | El-Sayed *et al.,* 2020 |
| MW528589 | Fusidium spp. (96.06) | Sordariomycete sp. A |  | Maharachchikumbura *et al*, 2016 |
| MW528590 | *Myrothecium chiangmaiense* (100) | Sordariomycete sp. B |  |  |
| MW528591 | *Tolypocladium album* (100) | *Tolypocladium album* | Animal Pathogen Endophyte Fungal Parasite | Hanada *et al.,* 2010  Gazis *et al.,* 2014 Fukuda *et al.,* 2015 |
| MW528592 | *Trichoderma koningii* (99.63) | *Trichoderma koningii* | Endophyte Plant Pathogen  Fungal parasite | Sankaranarayanan *et al.,* 1997  Xiao-Yan *et al.,* 2006  Worasatit *et al.,* 1994 Taha *et al*., 2021 |
|  |  |  |  |  |
|  |  |  |  |  |
| **Species not present in *B. rupestre*** | | | | |
| MW528593 | *Alfaria dandenongensis* (100) | *Alfaria dandenongensis* | Undefined | Lombard *et al.,* 2016 |
| MW528594 | *Dilophospora alopecuri* (99.76) | *Dilophospora* sp. | Plant Pathogen | Riley *et al*., 1998 Barbetti *et al*., 2006 |
| MW528595 | *Epichloë baconii* (100) | *Epichloë baconii* | Endophyte Plant pathogen | Romo-Vaquero *et al*., 2003 |
| MW528596 | *Epichloë festucae* (99.79) | *Epichloë festucae* | Endophyte Fungal parasite | Vázquez.de-Aldana *et al*., 2013 Zabalgogeazcoa et al., 2013 |
| MW528597 | *Paracremonium* sp. (88.59) | Nectriaceae sp. | Animal Pathogen Endophyte Fungal Parasite Lichen Parasite Plant Pathogen Wood Saprotroph | Lombard *et al*., 2015 Jumpponen *et al*., 2010 Zheng and Gong, 2019 |
| MW528598 | *Oculimacula anguioides* (97.29) | *Oculimacula* sp. | Plant Pathogen | Parnell *et al*., 2008 Vera and Murray, 2016 |
| MW528599 | *Scytalidium album* (99.78) | *Scytalidium album* | Wood Saprotroph | El-Elimat et al., 2015 |
| MW528600 | Tricholomataceae sp. (100) | Tricholomataceae sp. | Ectomycorrhizal Fungal Parasite | Campoamor and Molina, 2001 |

**Supplementary references**

An, Z., Siegel, M.R., Hollin, W., Tsai, H., Schmidt, D., and Schardl, C.L. (1993) Relationships among Non-*Acremonium* sp. fungal endophytes in five grass species. Applied and Environmental Microbiology 59, 1540–1548. doi: 10.1128/AEM.59.5.1540-1548.1993

Barbetti, M.J., and Riley, I.T. (2006) Field application of *Dilophospora alopecuri* to manage annual ryegrass toxicity caused by *Rathayibacter toxicus*. Plant Dis. 90:229-232. doi: 10.1094/PD-90-0229

Bezerra, J.D.P., Nascimento, C.C.F., Barbosa, R. do N., Da Silva, D.C.V., Svedese, V.M., Silva-Nogueira, E.B., Gomes, B.S., Paiva, L.M., and Souza-Motta, C.M. (2015) Endophytic fungi from medicinal plant *Bauhinia forficata*: Diversity and biotechnological potential. Brazilian Journal of Microbiology 46, 49–57. doi:10.1590/S1517-838246120130657

Campoamor, J.N., and Molina, J.A. (2001) Diversity of Tricholomataceae along a mediterranean altitudinal gradient. Cryptogamie mycologie 22 (3) 175-184. doi: 10.1016/S0181-1584(01)01068-5

Chen, C., Verkley, G.J.M., Sun, G., Groenewald, J.Z., and Crous, P.W. (2016) Redefining common endophytes and plant pathogens in *Neofabraea*, *Pezicula*, and related genera. Fungal Biology 120, 1291–1322. doi:10.1016/j.funbio.2015.09.013

Chi, W.C., Chen, W., He, C.C., Guo, S.Y., Cha, H.J., Tsang, L.M., Ho, T.W., and Pang, K.L. (2019) A highly diverse fungal community associated with leaves of the mangrove plant *Acanthus* *ilicifolius* var. *xiamenensis* revealed by isolation and metabarcoding analyses. PeerJ 2019. doi:10.7717/peerj.7293

Clarke, T.C., Shetty, K.G., Jayachandran, K., and Norland, M.R. (2007) *Myrothecium verrucaria* - A potential biological control agent for the invasive “old world climbing fern” (*Lygodium* *microphyllum*). BioControl 52, 399–411. doi:10.1007/s10526-006-9035-3

Clay, K., and Schardl, C. (2002) Evolutionary Origins and Ecological Consequences of Endophyte Symbiosis with Grasses. The American Naturalist 160, S99. doi:10.2307/3079271

Crous, P.W., Verkley, G.J.M., Christensen, M., Castañeda-Ruiz, R.F., and Groenewald, J.Z. (2012) How important are conidial appendages? Persoonia: Molecular Phylogeny and Evolution of Fungi 28, 126–137. doi:10.3767/003158512X652624

Crous, P.W., Wingfield, M.J., Burgess, T.I., Hardy, G.E.S.J., Crane, C., Barrett, S., Roux, J.J. Le, Thangavel, R., Guarro, J., Stchigel, A.M., Martín, M.P., Alfredo, D.S., Barber, P.A., Barreto, R.W., Baseia, I.G., Enwistle, P., Fiuza, P.O., Fournier, J., García, D., Gibertoni, T.B., and Giraud, S. (2016) Fungal Planet description sheets : 469 – 557. Persoonia 37, 218–403.

Dastogeer, K.M.G., Li, H., Sivasithamparam, K., Jones, M.G.K., and Wylie, S.J. (2018) Host Specificity of Endophytic Mycobiota of Wild Nicotiana Plants from Arid Regions of Northern Australia. Microbial Ecology 75, 74–87. doi:10.1007/s00248-017-1020-0

Doilom, M., Liu, J-K., Jaklitsch, W.M., Ariyawansa, H., Wijayawardene, N.N., Chukeatirote, E., Zhang, M., McKenzie, E.H.C., Geml, J., Voglmayr, H., and Hyde, K.D. (2013) An outline of the family Cucurbitariaceae. Sydowia 65, 164–192.

El-Elimat, T., Raja, H., Figueroa, M., Swanson, S.M., Falkinham, J.O., Lucas, D.M., Grever, M.R., Wani, M.C., Pearce, C.J., and Oberlies, N.H. (2015) Sorbicillinoid analogs with cytotoxic and selective anti-*Aspergillus* activities from *Scytalidium album*. The journal of antibiotics 68, 191-196. doi: 10.1038/ja.2014.125

El-Sayed, A.S.A., Moustafa, A.H., Hussein, H.A., El-Sheikh, A.A., El-Shafey, S.N., Fathy, N.A.M., and Enan, G.A. (2020) Potential insecticidal activity of *Sarocladium strictum*, an endophyte of *Cynanchum acutum*, against *Spodoptera littoralis,* a polyphagous insect pest. Biocatalysis and Agricultural Biotechnology 24, 101524. doi:10.1016/j.bcab.2020.101524

Ernst, M., Neubert, K., Mendgen, K.W., and Wirsel, S.G. (2011) Niche differentiation of two sympatric species of *Microdochium* colonizing the roots of common reed. BMC Microbiology 11. doi:10.1186/1471-2180-11-242

Fan, N.W., Chang, H.S., Cheng, M.J., Chan, H.Y., Hsieh, S.Y., Liu, T.W., Chen, S.W., Yuan, G.F., and Chen, I.S. (2016) New metabolites from the endophytic fungus *Mollisia* sp. Chemistry of Natural Compounds 52 (4). doi: 10.1007/s106000-016-1718-0

Flores, F.J., Marek, S.M., Anderson, J.A., Mitchell, T.K., and Walker, N.R. (2015) Infection and colonization of several bermudagrasses by *Ophiosphaerella korrae*. Phytopathology 105, 656–661. doi:10.1094/PHYTO-07-14-0205-R

Fournier, B., Pereira Dos Santos, S., Gustavsen, J.A., Imfeld, G., Lamy, F., Mitchell, E.A.D., Mota, M., Noll, D., Planchamp, C., and Heger, T.J. (2020) Impact of a synthetic fungicide (fosetyl-Al and propamocarb-hydrochloride) and a biopesticide (*Clonostachys rosea*) on soil bacterial, fungal, and protist communities. Science of the Total Environment 738, 1–10. doi:10.1016/j.scitotenv.2020.139635

Fukuda, T., Sudoh, Y., Tsuchiya, Y., Okuda, T., Matsuura, N., Motojima, A., Oikawa, T., and Igarashi, Y. (2015) Tolypoalbin, a new tetramic acid from *Tolypocladium album* TAMA 479. Journal of Antibiotics 68, 399–402. doi:10.1038/ja.2014.165

Jumpponen, A., Jones, K.L., and Blair, J. (2010) Vertical distribution of fungal communities in tallgrass prairie soil. Mycologia 102 (5) 1027-1041. doi: 10.3852/09-316

Gazis, R., Skaltsas, D., and Chaverri, P. (2014) Novel endophytic lineages of *Tolypocladium* provide new insights into the ecology and evolution of *Cordyceps*-like fungi. Mycologia 106, 1090–1105. doi:10.3852/13-346

Gross, A., Beenken, L., Dubach, V., Queloz, V., Tanaka, K., Hashimoto, A., and Holdenrieder, O. (2017) *Pseudodidymella fagi* and *Petrakia deviata*: Two closely related tree pathogens new to central Europe. Forest Pathology 47, 1–15. doi:10.1111/efp.12351

Hanada, R.E., Pomella, A.W. V., Costa, H.S., Bezerra, J.L., Loguercio, L.L., and Pereira, J.O. (2010) Endophytic fungal diversity in *Theobroma cacao* (cacao) and *T. grandiflorum* (cupuaçu) trees and their potential for growth promotion and biocontrol of black-pod disease. Fungal Biology 114, 901–910. doi:10.1016/j.funbio.2010.08.006

Hernández-Restrepo, M., Bezerra, J.D.P., Tan, Y.P., Wiederhold, N., Crous, P.W., Guarro, J., and Gené, J. (2019) Re-evaluation of *Mycoleptodiscus* species and morphologically similar fungi. Persoonia: Molecular Phylogeny and Evolution of Fungi 42, 205–227. doi:10.3767/persoonia.2019.42.08

Hernández-Restrepo, M., Groenewald, J.Z., Elliott, M.L., Canning, G., McMillan, V.E., and Crous, P.W. (2016a) Take-all or nothing. Studies in Mycology 83, 19–48. doi:10.1016/j.simyco.2016.06.002

Hernández-Restrepo, M., Groenewald, J.Z., and Crous, P.W. (2016b) Taxonomic and phylogenetic re-evaluation of *Microdochium*, *Monographella* and *Idriella*. Persoonia 36, 57-82. doi: 10.3767/003158516X688676

Hoagland, R.E., Boyette, C.D., and Abbas, H.K. (2007) *Myrothecium verrucaria* isolates and formulations as bioherbicide agents for kudzu. Biocontrol Science and Technology 17, 721–731. doi:10.1080/09583150701527268

Hou, G., Chen, X., Li, J., Ye, Z., Zong, S., and Ye, M. (2019) Physicochemical properties, immunostimulatory activity of the *Lachnum* polysaccharide and polysaccharide-dipeptide conjugates. Carbohydrate polymers 206: 446-454. doi: 0.1016/j.carbpol.2018.09.067

Inyang, E.N., Butt, T.M., Ibrahim, L., Clark, S.J., Pye, B.J., Beckett, A., and Archer, S. (1998) The effect of plant growth and topography on the acquisition of conidia of the insect pathogen *Metarhizium anisopliae* by larvae of *Phaedon cochleariae*. Mycological Research 102, 1365–1374. doi:10.1017/S095375629800673X

Jaklitsch, W.M., and Voglmayr, H. (2017) Three former taxa of *Cucurbitaria* and considerations on *Petrakia* in the Melanommataceae. Sydowia 69, 81–95. doi:10.12905/0380.sydowia69-2017-0081

Jie, C., Zhou, Q., Zhao, W., and Jiang, Y. (2013) A new *Myrmecridium* species from Guizhou, China. Mycotaxon 124, 1–8. doi: 10.5248/124.1

Kang, J.C., Hyde, K.D., and Kong, R.Y.C. (1999) Studies on Amphisphaeriales: The Amphisphaeriaceae (sensu stricto). Mycological Research 103, 53–64. doi:10.1017/S0953756298006650

Kornsakulkarn, J., Saepua, S., Suvannakad, R., Supothina, S., Boonyuen, N., Isaka, M., Prabpai, S., Kongsaeree, P., and Thongpanchang, C. (2017) Cytotoxic tropolones from the fungus *Nemania* sp. BCC 30850. Tetrahedron 73, 3505–3512. doi:10.1016/j.tet.2017.05.030

Krohn, K., Sohrab, M.H., Van Ree, T., Draeger, S., Schulz, B., Antus, S., and Kurtán, T. (2008) Dinemasones A, B and C - New bioactive metabolites from the endophytic fungus *Dinemasporium strigosum*. European Journal of Organic Chemistry 5, 5638–5646. doi:10.1002/ejoc.200800688

Lahlali, R., and Peng, G. (2014) Suppression of clubroot by *Clonostachys rosea* via antibiosis and induced host resistance. Plant Pathology 63, 447–455. doi:10.1111/ppa.12112

Liu, D., Sun, H., and Ma, H., 2019. Deciphering microbiome related to rusty roots of *Panax ginseng* and evaluation of antagonists against pathogenic *Ilyonectria*. Frontiers in Microbiology 10, 1–12. doi:10.3389/fmicb.2019.01350

Liu, J.M., Zhang, D.W., Du, W.Y., Zhang, M., Zhao, J.L., Chen, R.D., Xie, K.B., and Dai, J.G. (2017) Four new monoterpenoids from an endophytic fungus *Periconia* sp. F-31. Journal of Asian Natural Products Research 19, 541–549. doi:10.1080/10286020.2017.1313241

Lombard, L., Houbraken, J., Decock, C., Samson, R.A., Meijer, M., Réblová, M., Groenewald, J.Z., and Crous, P.W. (2016) Generic hyper-diversity in Stachybotriaceae. Persoonia: Molecular Phylogeny and Evolution of Fungi 36, 156–246. doi:10.3767/003158516X691582

Lombard, L., van der Merwe, N.A., Groenewald, J.Z., and Crous, P.W. (2015) Generic concepts in Nectriaceae. Studies in mycology 80: 189-245. doi: 10.1016/j.simyco.2014.12.002

Lomer, C.J., Bateman, R.P., Johnson, D.L., Langewald, J., and Thomas, M. (2001) Biological control of locusts and grasshoppers. Annual Review of Entomology 46, 667–702. doi:10.1146/annurev.ento.46.1.667

Lynch, S.C., Zambino, P.J., Mayorquin, J.S., Wang, D.H., and Eskalen, A. (2013) Identification of new fungal pathogens of coast live oak in California. Plant Disease 97, 1025–1036. doi:10.1094/PDIS-11-12-1055-RE

Maharachchikumbura, S.S.N., Hyde, K.D., Jones, E.B.G., *et al*., (2016) Families of Sordariomycetes, Fungal Diversity. doi:10.1007/s13225-016-0369-6

Manzanilla-López, R., and Lopez-Llorca, L.V. (2017) Perspectives in sustainable nematode management through Pochonia chlamydosporia applications for root and rhizosphere health. In: Peshin, R., Dhawan, A. (eds) Sustainability in plant crop protection. Springer. doi: 10.1007/978-3-319-99768-1Martínez-Álvarez, P., Fernández-González, R.A., Sanz-Ros, A.V., Pando, V., Diez, J.J. (2016) Two fungal endophytes reduce the severity of pitch canker disease in *Pinus radiata* seedlings. Biological Control 94, 1–10. doi:10.1016/j.biocontrol.2015.11.011

Martínez-Diz, M.P., Díaz-Losada, E., Armengol, J., León, M., Berlanas, C., Andrés-Sodupe, M., and Gramaje, D. (2018) First report of *Ilyonectria robusta* causing black foot disease of grapevine in Spain. Plant Disease 102, 2381. doi:10.1094/PDIS-05-18-0730-PDN

McMullin, D.R., Green, B.D., Prince, N.C., Tanney, J.B., and Miller, J.D. (2017) Natural Products of *Picea* Endophytes from the Acadian Forest. Journal of Natural Products 80, 1475–1483. doi:10.1021/acs.jnatprod.6b01157

Miller, S.L., Larsson, E., Larsson, K.-H., Verbeken, A., and Nuytinck, J. (2006) Perspectives in the new Russulales. Mycologia 98, 960–970. doi:10.1080/15572536.2006.11832625

Mousa, W.K., Schwan, A., Davidson, J., Strange, P., Liu, H., Zhou, T., Auzanneau, F.I., and Raizada, M.N. (2015) An endophytic fungus isolated from finger millet (*Eleusine coracana*) produces anti-fungal natural products. Frontiers in Microbiology 6:1154 doi:10.3389/fmicb.2015.01157

Newsham, K.K. (2011) A meta-analysis of plant responses to dark septate root endophytes. New Phytologist 190, 783–793. doi:10.1111/j.1469-8137.2010.03611.x

Ogura-Tsujita, Y., Gebauer, G., Hashimoto, T., Umata, H., and Yukawa, T. (2009) Evidence for novel and specialized mycorrhizal parasitism: The orchid *Gastrodia confusa* gains carbon from saprotrophic *Mycena*. Proceedings of the Royal Society: Biological Sciences 276, 761–767. doi:10.1098/rspb.2008.1225

Ortega, H.E., Graupner, P.R., Asai, Y., TenDyke, K., Qiu, D., Shen, Y.Y., Rios, I., Arnold, A.E., Coley, P.D., Kursar, T.O., Gerwick, W.H., and Cubilla-Rios, L. (2013) Mycoleptodiscins A and B, cytotoxic alkaloids from the endophytic fungus *Mycoleptodiscus* sp. F0194. Journal of Naturals Products 76, 741-744. doi: 10.1021/np300792t

Parnell, S., Gilligan, C.A., Bock, C.H., and van den Bosch, F. (2008) Changes in fungicide sensitivity and relative species abundance in *Oculimacula yallundae* and *O. acuformis* populations (eyespot disease of cereals) in Western Europe. Plant pathology 57, 509-517. doi: 10.1111/j.1365-3059.2007.01786.x

Pereira, E., Vázquez-De-Aldana, B., San Emeterio, L., and Zabalgogeazcoa, Í. (2019) A survey of culturable fungal endophytes from *Festuca rubra* subsp. *pruinosa*, a grass from marine cliffs, reveals a core microbiomes. Frontiers in Microbiology 9, 1–14. doi:10.3389/fmicb.2018.03321

Réblová, M., Barr, M.E., and Samuels, G.J. (1999) Chaetosphaeriaceae, a new family for *Chaetosphaeria* and its relatives. Sydowia 51, 49–70.

Riley, I.T., Reardon, T., and Bertozzi, T. (1998) Allozyme analysis of Australian isolates of *Dilophospora alopecuri.* Mycological research 102 (3), 301-307. doi: 10.1017/S095375629700498X

Rodriguez, M.A., Cabrera, G., Gozzo, F.C., Eberlin, M.N., and Godeas, A. (2011) *Clonostachys rosea* BAFC3874 as a *Sclerotinia sclerotiorum* antagonist: Mechanisms involved and potential as a biocontrol agent. Journal of Applied Microbiology 110, 1177–1186. doi:10.1111/j.1365-2672.2011.04970.x

Romo-Vaquero, M., Vázquez-De-Aldana, B.R., García-Ciudad, A., García-Criado, B., and Zalbalgogeazcoa, I. (2007) First report of choke disease caused by *Epichloë baconii* in the grass *Agrostis castellana*. Plant disease 90 (2) 314-314. doi: 10.1094/PDIS.2003.87.3.314B

Sankaranarayanan, C., and Hussaini, S.S. (1997) Nematicidal Effect of Fungal Filtrates against Root-Knot Nematodes. Journal of Biological Control 11, 37–41. doi:10.18311/jbc/1997/7575

Shadmani, L., Jamali, S., and Fatemi, A. (2020) Effects of root endophytic fungus, *Microdochium* *bolleyi* on cadmium uptake, translocation and tolerance by *Hordeum vulgare* L. Biologia. doi:10.2478/s11756-020-00598-5

Szarkándi, J.G., Schmidt-Stohn, G., Dima, B., Hussain, S., Kocsubé, S., Papp, T., Vágvölgyi, C., and Nagy, L.G. (2017) The genus *Parasola*: phylogeny and the description of three new species. Mycologia 109, 620–629. doi:10.1080/00275514.2017.1386526

Taha, M.A., Ismaiel, A.A., and Ahmed, R.M. (2021) 6-pentyl-α-pyrone from *Trichoderma koningii* induces systemic resistance in tobacco against tobacco mosaic virus. European Journal of Plant Pathology 159, 81-93. doi: 10.1007/s10658-020-02142-2

Tanney, J.B., and Seifert, K.A. (2020) Mollisiaceae: An overlooked lineage of diverse endophytes. Studies in Mycology 95, 293–380. doi:10.1016/j.simyco.2020.02.005

Tejesvi, M. V., Ruotsalainen, A.L., Markkola, A.M., and Pirttilä, A.M. (2010) Root endophytes along a primary succession gradient in northern Finland. Fungal Diversity 41, 125–134. doi:10.1007/s13225-009-0016-6

Tian, J., Lai, D., and Zhou, L. (2017) Secondary metabolites from *Acremonium* fungi: Diverse structures and bioactivities. Mini. Rev. Med. Chem. 17, 603–632. doi: 10.2174/1389557516666160914194134

Vázquez-de-Aldana, B.R., Zabalgogeazcoa, I., García-Ciudad, A., and García-Criado, B. (2013) An *Epichloë* endophyte affects the competitive ability of *Festuca rubra* against other grassland species. Plant soil 362: 201-213. doi: 10.1007/s11104-012-1283-7

Vera, D.I., Murray, T.D. (2016) Occurrence and survival of apothecia of the eyespot pathogens 10.1094/PDIS-09-15-1056-RE *Oculimacula acuformis* and *O. yallundae* on wheat stubble in the U.S. Pacific Northwest. Plant Dis. doi: 100:991-995.

Visagie, C.M., Houbraken, J., Seifert, K.A., Samson, R.A., and Jacobs, K. (2015) Four new *Penicillium* species isolated from the fynbos biome in South Africa, including a multigene phylogeny of section *Lanata-Divaricata*. Mycological Progress 14:96 doi:10.1007/s11557-015-1118-z

White, J.F.J. (1988) Endophyte-host associations in forage grasses. XI. A proposal concerning origin and evolution. Mycologya 80, 442–446. doi: 10.1080/00275514.1988.12025565

Worasatit, N., Sivasithamparam, K., Ghisalberti, E.L., and Rowland, C. (1994) Variation in pyrone production, lytic enzymes and control of *Rhizoctonia* root rot of wheat among single-spore isolates of *Trichoderma koningii.* Mycological Research 98 (12), 1357-1363. doi: 10.1016/S0953-7562(09)81063-0

Wu, M-L. (2003) A new species of *Albotricha* from Taiwan. Mycotaxon 88: 387-392.

Xiao-Yan, S., Qing-Tao, S., Shu-Tao, X., Xiu-Lan, C., Cai-Yun, S., and Yu-Zhong, Z. (2006) Broad-spectrum antimicrobial activity and high stability of Trichokonins from *Trichoderma* *koningii* SMF2 against plant pathogens. FEMS Microbiology Letters 260, 119–125. doi:10.1111/j.1574-6968.2006.00316.x

Xu, C., Li, J., Yang, Liuqing, Shi, F., Yang, Liu, and Ye, M. (2017) Antibacterial activity and a membrane damage mechanism of *Lachnum* YM30 melanin against *Vibrio* *parahaemolyticus* and *Staphylococcus aureus*. Food Control 73, 1445–1451. doi:10.1016/j.foodcont.2016.10.048

Youssar, L., Grüning, B.A., Erxleben, A., Günther, S., and Hüttel, W. (2012) Genome sequence of the fungus *Glarea lozoyensis*: The first genome sequence of a species from the helotiaceae family. Eukaryotic Cell 11, 250. doi:10.1128/EC.05302-11

Zabalgogeazcoa, I., Ciudad, A.G., Leuchtmann, A., Vázquez-De-Aldana, B.R., and Criado, B.G. (2008) Effects of choke disease in the grass *Brachypodium phoenicoides*. Plant Pathology 57, 467–472. doi:10.1111/j.1365-3059.2007.01784.x

Zabalgogeazcoa, I., Gundel, P.E., Helander, M., and Saikkonen, K. (2013) Non-systemic fungal endophytes in *Festuca rubra* plants infected by *Epichloë festucae* in subarctic habitats. Fungal diversity 60: 25-32. doi: 10.1007/s13225-013-0233-x

Zhang, W., Krohn, K., Draeger, S., and Schulz, B. (2008) Bioactive isocoumarins isolated from the endophytic fungus *Microdochium* *bolleyi*. Journal Natural Products 71: 1078-1081. doi: 10.1021/np800095g

Zheng, Y., and Gong, X. (2019) Niche differentiation rather than biogeography shapes the diversity and composition of microbiome of *Cycas panzhihuaensis*. Microbiome 7:152. Doi: 10.1186/s40168-019-0770-y

**Supplementary material, table S2:** The presence/absence data of the *B. rupestre* identified taxa.

| **Fungal endophyte** | ***B.rupestre* tissue** | | | **Low-diversity grasslands** | | | **High-diversity grasslands** | | |
| --- | --- | --- | --- | --- | --- | --- | --- | --- | --- |
|  |  |  |  |  |  |  |  |  |  |
|  | **Shoot** | **Rhizome** | **Root** | **Arpea** | **Errozate** | **Armorieta** | **Urkulu** | **Zalbetea** | **Azalegi** |
| *Acremonium* sp. | 0 | 0 | 4 | 1 | 0 | 1 | 2 | 0 | 0 |
| *Albotricha* sp. | 0 | 20 | 33 | 1 | 3 | 9 | 23 | 17 | 0 |
| *Alfaria thymi* | 0 | 1 | 0 | 0 | 0 | 0 | 0 | 1 | 0 |
| Amphisphaeriaceae sp. | 0 | 0 | 1 | 0 | 0 | 1 | 0 | 0 | 0 |
| *Bullanockia* sp. | 0 | 0 | 1 | 0 | 0 | 1 | 0 | 0 | 0 |
| *Cadophora* sp. | 0 | 0 | 1 | 0 | 0 | 0 | 0 | 0 | 1 |
| *Chaetosphaeria* sp. | 0 | 0 | 2 | 1 | 0 | 1 | 0 | 0 | 0 |
| Chaetosphaeriales sp. | 0 | 0 | 1 | 0 | 0 | 0 | 0 | 0 | 1 |
| *Clonostachys rosea* | 0 | 4 | 0 | 0 | 2 | 0 | 1 | 1 | 0 |
| Cucurbitariaceae sp. | 0 | 2 | 1 | 2 | 0 | 0 | 0 | 1 | 0 |
| *Dictyochaeta* sp. | 0 | 0 | 4 | 2 | 0 | 2 | 0 | 0 | 0 |
| *Dinemasporium morbidum* | 1 | 0 | 0 | 0 | 0 | 0 | 1 | 0 | 0 |
| *Epichloe typhina* | 7 | 2 | 2 | 0 | 0 | 4 | 3 | 4 | 0 |
| *Fusarium circinatum* | 0 | 5 | 4 | 1 | 1 | 1 | 0 | 2 | 4 |
| *Gaeumannomycella* sp. | 0 | 2 | 0 | 2 | 0 | 0 | 0 | 0 | 0 |
| *Glarea* sp. | 0 | 2 | 6 | 1 | 0 | 2 | 0 | 4 | 1 |
| Hypocreales sp. | 0 | 1 | 0 | 0 | 0 | 1 | 0 | 0 | 0 |
| *Ilyonectria robusta* | 0 | 3 | 7 | 0 | 2 | 1 | 0 | 5 | 2 |
| *Lachnum* sp. A | 0 | 1 | 9 | 4 | 1 | 1 | 0 | 3 | 1 |
| *Lachnum* sp. B | 1 | 24 | 59 | 14 | 26 | 14 | 9 | 4 | 17 |
| *Metapochonia bulbillosa* | 0 | 6 | 0 | 3 | 0 | 0 | 1 | 1 | 1 |
| *Metarhizium carneum* | 3 | 0 | 0 | 2 | 0 | 1 | 0 | 0 | 0 |
| *Microdochium bolleyi* | 0 | 3 | 2 | 1 | 0 | 0 | 0 | 1 | 3 |
| *Microdochium neoqueenslandicum* | 0 | 0 | 2 | 0 | 0 | 1 | 0 | 0 | 1 |
| *Mollisia* sp. | 0 | 0 | 3 | 2 | 0 | 1 | 0 | 0 | 0 |
| Mollisiaceae sp. | 3 | 23 | 33 | 10 | 18 | 6 | 8 | 7 | 10 |
| *Mycena* sp. | 0 | 1 | 0 | 0 | 0 | 0 | 1 | 0 | 0 |
| *Myrmecridium* sp. | 1 | 0 | 0 | 0 | 0 | 0 | 0 | 1 | 0 |
| *Myrothechium* sp. | 0 | 1 | 0 | 0 | 0 | 1 | 0 | 0 | 0 |
| *Nemania* sp. | 0 | 1 | 0 | 1 | 0 | 0 | 0 | 0 | 0 |
| *Omnidemptus graminis* | 80 | 12 | 0 | 19 | 20 | 20 | 12 | 15 | 6 |
| *Ophiospaerella korrae* | 0 | 0 | 1 | 0 | 0 | 0 | 0 | 0 | 1 |
| *Parasola* sp. | 0 | 1 | 0 | 1 | 0 | 0 | 0 | 0 | 0 |
| *Penicillium ortum* | 0 | 5 | 2 | 0 | 1 | 1 | 0 | 0 | 5 |
| Peniophoraceae sp. | 1 | 0 | 0 | 0 | 0 | 1 | 0 | 0 | 0 |
| *Periconia macrospinosa* | 0 | 1 | 0 | 1 | 0 | 0 | 0 | 0 | 0 |
| *Petrakia* sp. | 0 | 0 | 1 | 0 | 0 | 0 | 0 | 0 | 1 |
| *Pezicula* sp. | 0 | 0 | 1 | 0 | 0 | 0 | 0 | 1 | 0 |
| Pleosporales sp. | 0 | 0 | 1 | 0 | 0 | 0 | 0 | 0 | 1 |
| *Pyrenochaetopsis leptospora* | 0 | 0 | 1 | 0 | 0 | 0 | 0 | 1 | 0 |
| *Sarocladium strictum* | 4 | 0 | 0 | 1 | 3 | 0 | 0 | 0 | 0 |
| Sordariomycete sp. A | 0 | 0 | 1 | 0 | 0 | 0 | 0 | 1 | 0 |
| Sordariomycete sp. B | 0 | 1 | 0 | 0 | 0 | 0 | 1 | 0 | 0 |
| *Tolypocladium album* | 0 | 4 | 0 | 0 | 0 | 3 | 1 | 0 | 0 |
| *Trichoderma koningii* | 0 | 1 | 0 | 1 | 0 | 0 | 0 | 0 | 0 |

**Supplementary material, table S3:** The presence/absence data of the aerial tissues in three representative grasses of high-diversity grasslands.

| **Fungal endophyte** | **Grass species** | | | **High-diversity grasslands** | | |
| --- | --- | --- | --- | --- | --- | --- |
|  |  |  |  |  |  |  |
|  | ***B. rupestre*** | ***F. rubra*** | ***A. capillaris*** | **Urkulu** | **Zalbetea** | **Azalegi** |
| *Albotricha* sp. | 0 | 4 | 2 | 2 | 3 | 1 |
| *Alfaria dandenongensis* | 0 | 3 | 3 | 5 | 1 | 0 |
| *Cadophora* sp. | 0 | 1 | 0 | 0 | 0 | 1 |
| *Clonostachys rosea* | 0 | 1 | 0 | 1 | 0 | 0 |
| *Dilophospora* sp. | 0 | 0 | 1 | 0 | 1 | 0 |
| *Dinemasporium morbidum* | 1 | 0 | 0 | 1 | 0 | 0 |
| *Epichloe baconii* | 0 | 0 | 5 | 3 | 0 | 2 |
| *Epichloe festucae* | 0 | 29 | 0 | 9 | 0 | 20 |
| *Epichloe typhina* | 4 | 0 | 0 | 1 | 3 | 0 |
| *Fusarium circinatum* | 0 | 0 | 1 | 0 | 0 | 1 |
| *Glarea* sp. | 0 | 0 | 1 | 0 | 1 | 0 |
| *Ilyonectria robusta* | 0 | 0 | 2 | 1 | 0 | 1 |
| *Lachnum* sp. B | 1 | 1 | 6 | 8 | 0 | 0 |
| *Microdochium neoqueenslandicum* | 0 | 1 | 1 | 1 | 0 | 1 |
| Mollisiaceae sp. | 0 | 9 | 5 | 13 | 0 | 1 |
| *Myrmecridium* sp. | 1 | 0 | 0 | 0 | 1 | 0 |
| Nectriaceae sp. | 0 | 0 | 1 | 0 | 1 | 0 |
| *Oculimacula* sp. | 0 | 0 | 1 | 0 | 1 | 0 |
| *Omnidemptus graminis* | 27 | 2 | 4 | 10 | 17 | 6 |
| *Pyrenochaetopsis leptospora* | 0 | 0 | 1 | 0 | 0 | 1 |
| *Scytalidium album* | 0 | 0 | 1 | 0 | 1 | 0 |
| Sordariomycete sp. B | 0 | 1 | 0 | 1 | 0 | 0 |
| Tricholomataceae sp. | 0 | 0 | 1 | 0 | 1 | 0 |


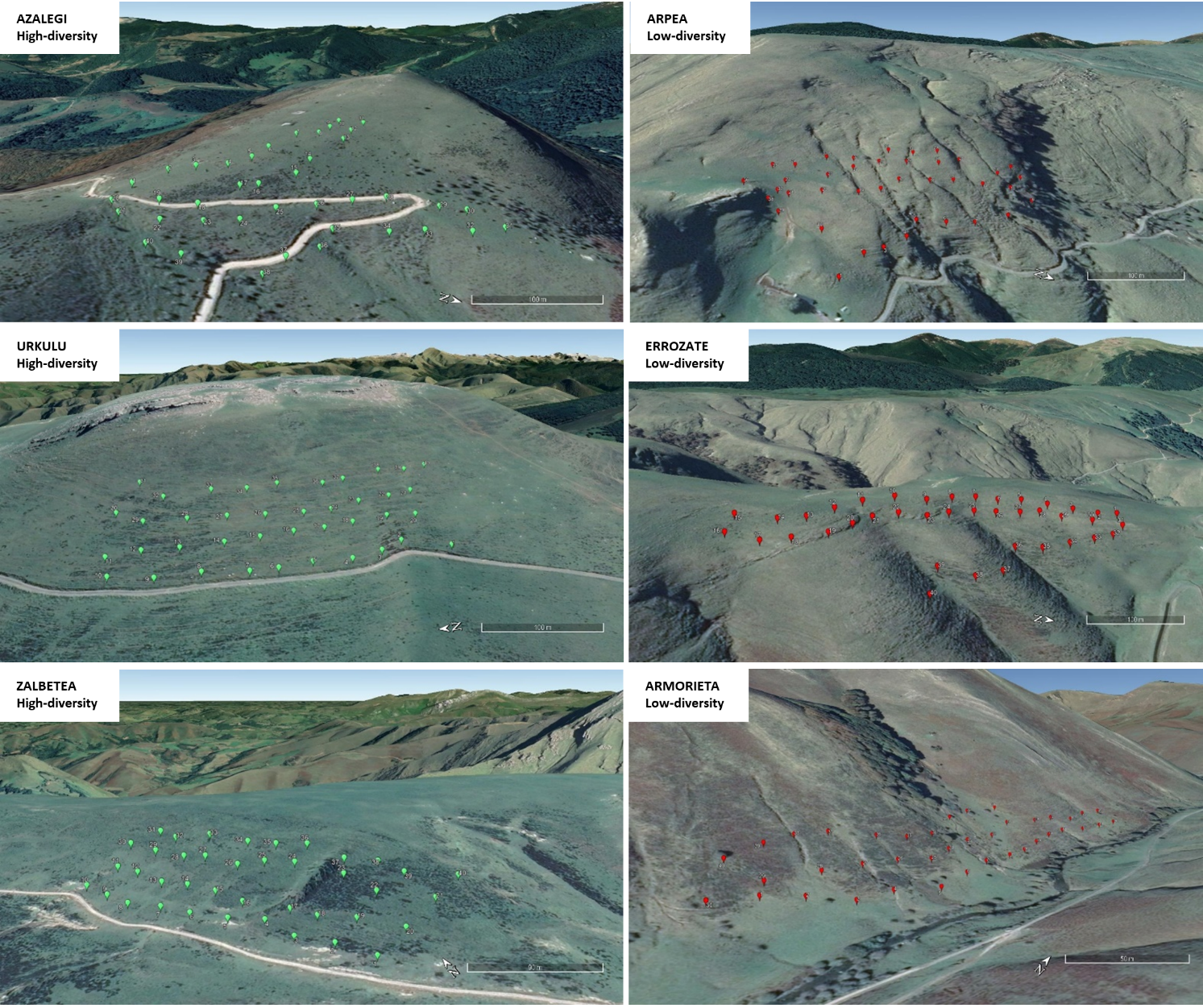


**Supplementary Figure S1:** Georeferenced sampling points in each location.
